# Two-Dimensional C_60_ Fullerene Networks (Graphullerene): Structure–Property Relations, Quantitative Benchmarking and Future Directions

**DOI:** 10.3390/nano16211385

**Published:** 2026-10-28

**Authors:** Giorgos S. Avntoulla, Georgios I. Giannopoulos

**Affiliations:** Department of Mechanical Engineering, Polytechnic School, University of the Peloponnese, GR-26334 Patras, Greece; gavntoulla@gmail.com

**Keywords:** graphullerene, polymeric C_60_, two-dimensional fullerene network, quasi-hexagonal phase, quasi-tetragonal phase, mechanical normalisation, thermal transport, excitons, tribology, photocatalysis

## Abstract

Two-dimensional covalent C_60_ networks are molecularly derived carbon allotropes in which intact fullerene cages form the repeating units of an extended lattice. Since isolation of a monolayer network in 2022, the field has expanded from synthesis to fracture, transport, excitonics, tribology, membranes, catalysis and energy storage. Growth has outpaced comparability: nominally identical quantities can refer to different structures, methods or dimensional conventions. Using literature verified through to 9 September 2026, we review the field within an evidence-graded framework and restate recoverable mechanical results on a thickness-independent basis. For the quasi-hexagonal phase, moduli published in GPa span 49.6–218.5 GPa (~4.4-fold), whereas the recoverable/native set occupies 93–189 N m^−1^ (~2.0-fold). Because the populations are not identical, this is a benchmarking diagnostic rather than an effect size; the narrower recoverable range is consistent with thickness conventions contributing to the apparent scatter. Thermal transport remains less settled: the direct experimental benchmark (~2.7 W m^−1^ K^−1^) is for micrometre-thick layered material, while monolayer simulations span a few to >100 W m^−1^ K^−1^. Electronic studies establish a semiconducting, anisotropic platform, although reported gaps remain observable- and method-dependent. We conclude with reporting standards and research priorities for comparable C_60_-network studies.

## 1. Introduction

The experimental conversion of fullerene cages from discrete molecular units into covalently connected two-dimensional solids created a carbon architecture that is neither a conventional molecular crystal nor an atomically flat sp^2^ sheet. In the target materials reviewed here, the C_60_ cage remains recognisable, but neighbouring cages are joined through directional covalent bridges. The resulting lattice inherits molecular curvature and internal cage modes while simultaneously acquiring extended electronic bands, in-plane elasticity and collective phonons. This mixed molecular/crystalline character is the physical reason that graphullerene behaves differently from both fullerite and graphene [1,2,3,4].

The field has expanded rapidly since the first isolated covalent monolayer. Early work focused on structural identification and electronic transport; subsequent studies addressed fracture, thermal transport, excitonics, tribology, membranes, catalysis and scalable processing. Recent experiments extend the application space to sodium storage and conversion and show that coordination chemistry can cleave selected inter-cage bonds and release intact C_60_, introducing a route toward chemically programmable disassembly [5,6].

This shift is easier to understand when placed against the dimensional hierarchy of the carbon allotropes. Diamond is the three-dimensional sp^3^ network, graphite a van der Waals stack of sp^2^ sheets, graphene the isolated sheet, the nanotube its rolled one-dimensional form, and the fullerene the closed zero-dimensional cage [1,2]. Each of these is built from atoms. A covalent C_60_ network is built from cages: the repeating unit is itself a sixty-atom molecule with internal structure, curvature and its own vibrational spectrum. This is why the material does not behave simply as an additional two-dimensional carbon. Properties that in graphene are set by a two-atom basis are here set by a hierarchy—intra-cage bonding, inter-cage bridging, and, in multilayers, interlayer registry—and each level can be varied independently.

The consequences are visible across every property class. Mechanically, deformation proceeds not only by bond stretching but by cage rotation and junction distortion, so the elastic response is governed by the topology of the bridges, not by the lattice symmetry. Electronically, the closed-shell frontier orbitals of the molecule broaden into narrow, anisotropic bands, giving the experimentally established quasi-hexagonal phase (qHP) network a finite gap that graphene lacks. Thermally, the separation between low-frequency inter-cage modes and high-frequency intra-cage vibrations suppresses conduction far below graphene and many high-conductivity extended sp^2^ carbon networks. These are not independent observations but three consequences of the same architectural choice, and organising them around that common cause is one of the aims of this review.

This growth creates a methodological problem for a review article. Values that look contradictory may refer to different C_60_ bonding patterns, different axis conventions, different layer counts, different force fields, different temperature and strain-rate regimes, or different definitions of thickness. Conversely, values that numerically agree may not represent the same observable. A review that only catalogues reported numbers therefore risks producing false comparisons. The central aim here is to establish which comparisons are physically legitimate and which require re-expression, qualification or exclusion.

Several recent reviews have already mapped important parts of the field. Peng and Pizzochero surveyed monolayer networks from a first-principles perspective [7]; Wang et al. provided a broad synthesis-theory-applications overview [8]; Lv et al. reviewed covalent fullerene networks across dimensionalities with emphasis on preparation and catalysis [9]; and Paupitz et al. placed fullerene networks in the wider context of recently synthesised two-dimensional carbon allotropes [10]. The present article is intentionally different. Its purpose is quantitative benchmarking: it assigns evidence classes, distinguishes experimentally realised phases from hypothetical structures, normalises 2D mechanical quantities, exposes unresolved thermal-transport discrepancies, and proposes reporting conventions that make future papers mutually comparable. Figure 1 fixes the structural vocabulary used throughout, contrasting an isolated C_60_ cage with the two connectivity classes that recur in the literature.

## 2. Scope, Terminology and Literature Methodology

Two scope boundaries were available for this review, and the narrower one was adopted deliberately. The broader class is everything that has been called a two-dimensional fullerene material. It takes in covalently bridged C_60_ monolayers, non-covalent ordered cage arrays, metal-containing polymeric precursors, exfoliated fullerite platelets, and porous lattices assembled from smaller cages such as C_36_ or C_24_ [11]. The narrower class, adopted here as the quantitative core, is the subset in which intact C_60_ cages are joined to one another by in-plane covalent C–C bridges. That boundary was chosen because the structure–property relations benchmarked here are properties of that connectivity. Members of the broader class share no common in-plane load path, band structure or phonon spectrum, so pooling them would erase the distinctions being measured. Excluded members are not discarded but retained as labelled boundary cases at the two ends of the bonding scale, as Section 2.1 sets out. The literature search and verification for this revision was completed on 9 September 2026. This is a structured critical narrative review rather than a systematic review. The target class was defined by chemistry, not by dimensionality alone: a study was included as core literature when it addressed a two-dimensional or few-layer material in which C_60_ cages are covalently connected within the extended network. Search concepts included graphullerene, 2D fullerene network, polymeric C_60_ monolayer, qHP/qTP C_60_, quasi-hexagonal fullerene, quasi-tetragonal fullerene, fullerene nanosheet, covalent fullerene network, and property-specific combinations for mechanics, thermal transport, electronic structure, optics, catalysis, membranes, storage and tribology. Publisher pages, DOI metadata, citation tracing from core primary articles and recent field reviews were used to resolve ambiguous or duplicated citations and to identify new work. The methodology follows the conventions of a structured critical narrative review rather than those of a systematic review, and the distinction is stated explicitly because the two are held to different reporting standards. PRISMA-type systematic-review reporting is deliberately not claimed: no pooled meta-analysis, effect-size estimation or risk-of-bias scoring is performed, and the corpus is not exhaustive by construction. Four elements normally associated with systematic reporting are nevertheless adopted, because the central activity of this review is quantitative comparison across heterogeneous studies. The search concepts are declared above and the verification cut-off date is stated; the inclusion and exclusion rules are given before any evidence is presented (Section 2.1); and every entry in the corpus is classified by evidential proximity to the target material at result level rather than per paper (Section 2.2 and Table 1). In its scoping, the review follows the earlier first-principles perspective on this field [7] in treating the experimentally realised quasi-hexagonal monolayer as the reference structure, and departs from it in normalising every reported mechanical value to a thickness-independent form before any comparison is drawn (Section 6.3).

A critical scope is necessary here for a reason that does not arise in most review topics. The term graphullerene, and the looser phrase two-dimensional fullerene network, have been applied to structurally non-identical objects: covalently bridged monolayers of the quasi-hexagonal phase, few-layer and bulk polymerised phases in which interlayer bonding is also present, metal-containing precursors such as Mg_4_C_60_ before demetallation, non-covalent ordered arrays of intact cages, and porous lattices built from smaller cages such as C_36_ or C_24_. These differ not marginally but qualitatively: the connectivity that produces the anisotropic band structure and the order-of-magnitude stiffness increase is present in some and absent in others. A property value transferred between them without comment is not a comparison but a category error, and several of the disagreements catalogued in this review originate there rather than in the calculations themselves.

The inclusion rules below are therefore drawn around connectivity, not around nomenclature or dimensionality. Systems in which intact C_60_ cages are joined by in-plane covalent C–C bridges form the quantitative core. Systems in which the cages are present but the coupling is van der Waals, and systems in which the cage is destroyed or replaced by a different molecule, are retained only as reference points at the two ends of the bonding scale. Between those limits sit the supported, encapsulated, metal-decorated and chemically functionalised variants, which are included but never merged with freestanding results, because their reported properties are properties of an assembly. The graphene encapsulation results are the clearest illustration: they are computed for exactly the supported geometry a device would have, and they are also three to four times the freestanding stiffness [12], so averaging them into an intrinsic figure would corrupt it.

The core corpus that results is tabulated study by study in Appendix A, with the method and evidence class of each entry. Two limitations of the search should be stated. First, the field is moving fast enough that any cut-off is arbitrary; work appearing after the stated date is necessarily absent, and the 2026 entries included here were verified individually against publisher records and not accepted from secondary citation. Peer-reviewed papers without a version of record by the cut-off were not counted as published core literature, and retracted articles were excluded from the quantitative synthesis. Second, the quantitative core is dominated by computation. This is not a selection artefact but a property of the field: at the time of writing there is no direct measurement of the in-plane elastic modulus, the intrinsic strength or the thermal conductivity of an isolated covalent C_60_ monolayer. Accordingly, the intrinsic monolayer mechanical values and the available monolayer thermal-conductivity values in the comparison are model-derived; the broader tables also contain experimental bulk, multilayer and device benchmarks, which are labelled separately. The resulting corpus supports a transparent critical synthesis, but it should not be interpreted as an exhaustive systematic-review dataset.

### 2.1. Inclusion and Exclusion Rules

- Included as core evidence: monolayer, bilayer, trilayer or few-layer C_60_ networks with in-plane covalent C_60_–C_60_ connectivity, whether experimentally realised or computationally proposed.
- Included but labelled separately: supported, encapsulated, metal-decorated, chemically functionalised and heterostructured derivatives, because their measured properties are composite/interface properties, not pristine-network properties.
- Retained only as benchmarks: isolated C_60_, fullerite and classical polymerised bulk C_60_, which establish molecular/crystal baselines but are not themselves 2D graphullerene.
- Excluded from the quantitative core: non-covalent C_60_ arrays or nanosheets (including fcc molecular nanosheets used only as catalyst supports), molecular fullerene dyads, generic fullerene drug-adsorption systems, graphene defect literature and non-C_60_ fullerene networks unless required explicitly as a boundary comparison. The words ‘nanosheet’ or ‘2D fullerene’ alone were therefore not treated as evidence of a covalent C_60_ network.

### 2.2. Evidence Classes and Comparison Rules

The evidence class matters because the same word—‘graphullerene’—has been used for structurally non-identical models. In particular, the qTP label is not guaranteed to denote the same bond topology in Peng’s phase set [13], in later five-phase surveys [14], and in atomistic-force-field studies. Therefore, the primary identifiers for comparison are the exact inter-cage bond motif, molecular orientation, unit cell and coordination, not the phase abbreviation alone.

The classes used here measure evidential proximity to the target C_60_ network. They do not rank computational methods against one another. Class A denotes direct experimental evidence on pristine covalent C_60_ networks, including synthesis and intrinsic or near-intrinsic characterisation. Class B denotes experimental devices, supported materials, chemically functionalised sheets, metal-decorated networks and other derivatives whose measured response includes an interface or added component. Class C denotes theory or simulation performed on an experimentally anchored C_60_-network topology, irrespective of whether the method is first-principles, classical molecular dynamics or a machine-learned potential. Class D denotes theoretical work on an unrealised network topology, chemical derivative or functionality for which the corresponding material has not been established experimentally. Class E is reserved for molecular/bulk benchmarks, reviews and perspectives used for context, not as direct property evidence. A slash label such as A/B or C/D is used when one study reports distinct results belonging to more than one class; conclusions are assigned at result level. Table 1 lists the classes and the weight each one carries.

Conflicts between evidence classes are resolved by asking what each experiment or model is actually entitled to determine. Where a first-principles elastic constant and a reactive-molecular-dynamics modulus disagree for the same anchored topology, the comparison becomes informative only after the thickness convention is removed and the temperatures and loading definitions are matched; the residual difference then reflects the model and protocol. Where computed thermal conductivities disagree, neither result should be preferred solely because of the label attached to the method, and both should remain visible until experiment or a controlled cross-method benchmark resolves the discrepancy. Where computation conflicts with an experimental structural determination, the experimentally established structure sets the reference, which is why the quasi-hexagonal monolayer is treated throughout as the benchmark phase despite not being the lowest-energy candidate in several screening studies [7,13,15].

**Reporting rule 1.** Every quoted property should be accompanied by phase/topology, layer count, direction, temperature, computational or experimental method, and—when GPa or W m^−1^ K^−1^ is derived from a nominal 2D sheet—the thickness convention used to convert an areal quantity into a volumetric one.

## 3. Structural Taxonomy and Bonding

C_60_ contains 60 carbon atoms arranged in a truncated-icosahedral cage. Within a covalent 2D network, most cage atoms retain predominantly sp^2^ character, while atoms at inter-cage junctions become locally rehybridised as new sigma bonds form. Two recurrent connection motifs are direct C–C links and [2+2]-cycloaddition-derived bridges. Their number, orientation and distribution determine the effective symmetry felt by mechanical, electronic and phonon excitations [13,16,17,18,19,20,21].

That cage is more constrained than the atom count suggests, and the constraint is worth making explicit because later sections inherit it. The sixty atoms are all equivalent under the full Ih point group, arranged in twelve pentagons and twenty hexagons, with only two distinct bond lengths: the shorter bonds shared by two hexagons carry more double-bond character than the longer ones shared by a pentagon and a hexagon. That symmetry is among the highest realised in molecular chemistry, and it is why the cage behaves as a reproducible unit, not as an arbitrary carbon cluster—every network built from it starts from an identical node.

Its stability follows from the isolated pentagon rule: a fullerene is particularly stable when each of its twelve pentagons is completely surrounded by hexagons, so that no two pentagons share an edge and the strain associated with adjacent pentagons is avoided [22]. C_60_ is the smallest fullerene that satisfies this rule and is a dominant product of many high-temperature carbon-condensation routes, which helped make it the practical building block of this materials family. The rule also frames what polymerisation costs: forming an inter-cage bridge rehybridises the carbon atoms at that site from sp^2^ toward sp^3^ and perturbs a structure that was, in isolation, near a strain optimum. The pre-strained cage is therefore not a neutral node in the lattice but a unit that resists the geometry the lattice imposes on it, and the resulting distribution of bond lengths is a structural fingerprint that distinguishes network phases.

Two bridge motifs dominate the covalent networks, and their difference is the origin of most of the anisotropy discussed later. The [2+2] cycloaddition bridge joins two cages through a four-membered carbon ring, converting four cage atoms to a local sp^3^ geometry and producing a comparatively stiff, short and geometrically rigid link. The single C–C bridge joins the cages through one bond, is longer, and permits far more rotational freedom about its axis. A lattice built entirely from one motif behaves very differently under load from one built from a mixture, and the quasi-hexagonal phase is precisely a mixture: it combines single inter-cage bonds along one lattice direction with [2+2] bridges along another. The directional stiffness contrast therefore originates primarily in bond topology and is reinforced by molecular orientation and lattice geometry, which is why a crystallographic label alone cannot predict it.

The consequences propagate. For the experimentally anchored qHP orientation used in the cited models, when tension is applied along the direction dominated by [2+2] bridges, the response is close to bond stretching and is stiff and nearly harmonic; along the single-bond direction, angular bending and cage rotation are activated first, giving a softer and more non-linear response and, at failure, a different crack path [18,21]. Because the two motifs also differ in how much they perturb the cage π system, the same asymmetry appears in the electronic dispersion and in the optical absorption, which is why mechanical and optical anisotropy in these networks share an axis. Thermal expansion follows the same logic with opposite sign along the two motifs, expanding along the cycloaddition direction and contracting along the single-bond direction [23]. Few materials expose a single structural variable that controls four property classes so directly.

The design space extends beyond pristine C_60_ without requiring the cage framework to be abandoned. Networks built from smaller cages such as C_36_ have been predicted with different bond densities, stiffness and thermal behaviour [11], although such systems are retained here only as boundary comparisons. Within C_60_ itself, substitutional chemistry has now been explored in several directions. Boron nitride (BN) co-doping has been studied in both qTP-like and qHP sheets [24,25], while B, N and H impurities have been proposed as a route to spin-dependent electronic states [26]. Endohedral encapsulation of N, Ce or Sr provides a distinct form of tuning because the guest is placed inside the cage rather than substituted into the carbon framework [27]. Covalent chemistry on the sheet supplies a further axis: aryl-diazonium functionalisation attaches molecular groups to the exposed cage surface without destroying the network [28], and metal clusters can be anchored at intrinsic pore or hole sites between cages [29,30]. For the C_60_ core literature, the principal variables are therefore bridge topology, layer number/stacking and controlled chemical modification.

The quasi-hexagonal phase (qHP) is the experimentally established monolayer benchmark. In structural models matched to experiment, each cage is connected to six neighbours through an anisotropic mixture of bridge types, not through six equivalent atomic bonds [3,20]. The 2022 synthesis also reported few-layer qTP C_60_, so the tetragonal family is not purely theoretical; what remains uncertain is whether the various qTP, qTP1, qTP2 and TP labels used in later calculations and experiments describe identical bond topologies [3,13,14]. This distinction became even more important in 2026, when Lv et al. experimentally prepared hexagonal-phase and tetragonal-phase 2D-C_60_ nanosheets [31]. Their TP material should not be assumed to be structurally identical to a particular theoretical qTP monolayer without a bond-by-bond mapping. Peng’s 2023 analysis separated qTP1 and qTP2 and found sharply different stability and elasticity [13]. In contrast, Yan et al. later examined TP, TP2, qTP, qHP and RP patterns and found all five dynamically and mechanically stable within their own optimised models [14].

Mortazavi expanded the design space beyond the qHP/qTP dichotomy by screening many C_60_-based networks with different porosity and connectivity [15]. This predicted landscape includes metallic, semimetallic and semiconducting structures, and selected porous networks with mechanical metrics that can exceed dense phases under the same computational framework. Kayley and Peng later examined pressure-derived layered C_60_ crystals with in-plane [2+2] bonding and van der Waals interlayer coupling, showing that molecular orientation and crystal symmetry can tune lattice dynamics, electronic gaps and optical response [32]. These bulk or quasi-2D phases are useful structural benchmarks, but they belong outside the isolated qHP-monolayer property set. Likewise, hypothetical porous monolayers remain class-D predictions until experimentally realised.

Across these taxonomic studies, one conclusion is consistent, while another remains open. They agree that a crystallographic label such as hexagonal or tetragonal is insufficient, because the type, orientation and density of the inter-cage bonds, and not the arrangement of cage centres alone, fix the symmetry that is physically operative. Two lattices with similar cage packing but different bridge chemistry can behave differently under load and in transport. They diverge on what should therefore be ranked. Stability-based analyses can identify mechanically robust structures that are not thermodynamic minima [13], while broader energy screening returns many candidate lattices with closely spaced energies [15]. The available evidence therefore suggests that precursor geometry, kinetic accessibility and processing history play a major role alongside energetics. qHP remains the established monolayer reference; qTP/TP materials have been obtained in few-layer or nanosheet form [3,31], but structural equivalence to the various theoretical qTP variants should be demonstrated rather than inferred from the phase label.

The boundary of this material class becomes clearer when viewed from outside the covalent-network core. Ordered one-dimensional C_60_ arrays can be assembled within molecular host networks purely by non-covalent interactions: the cages are periodic and low-dimensional, yet no inter-cage covalent bond forms and the solid retains essentially molecular electronic behaviour. Neither dimensionality nor long-range order is therefore the defining criterion for this material class; covalent inter-cage connectivity is. The contrast isolates the variable that matters, since the transition from molecular crystal to extended anisotropic semiconductor tracks the formation of the bridges and not the ordering of the cages.

**Key structural insight.** Crystallographic labels are useful shorthand, but they are not sufficient identifiers. For C_60_ networks the mechanically and electronically relevant symmetry is set by the orientation and chemistry of inter-cage bridges; merging structures solely because they share the label “qHP” or “qTP” can create artificial disagreement.

## 4. Synthesis and Characterisation: From Isolated Flakes to Scalable Processing

Classical C_60_ production by laser vaporisation and bulk fullerene preparation established the molecular precursor platform [1,2], but it did not determine the topology of a 2D covalent network. The decisive advance came from constructing an ordered magnesium-containing polymeric fullerene precursor and selectively removing the interlayer component while preserving in-plane covalent order. Hou et al. thereby isolated a monolayer qHP C_60_ network with anisotropic semiconducting transport and, in the same study, reported few-layer qTP C_60_ [3]. Meirzadeh et al. subsequently demonstrated another few-layer covalent fullerene network, clarifying that the new bonding hierarchy survives beyond the monolayer limit [4].

The classical routes remain the source of essentially all C_60_ used as a precursor, and their limitations shape what follows. Arc-discharge vaporisation of graphite in a sub-atmospheric inert atmosphere produces the soot from which solid C_60_ is extracted [2], and the same cage forms in the gas phase under laser vaporisation into a helium flow [1]. Growth itself was established later: exposing fullerenes to carbon vapour from ^13^C-enriched targets and following the products by Fourier-transform ion cyclotron resonance mass spectrometry shows that fullerenes self-assemble by closed-network growth, incorporating atomic carbon and C_2_ into an already closed cage, and that the bond rearrangements accompanying growth are carbon-catalysed rather than Stone–Wales, whose barrier is prohibitive at 164.6 against 58.5 kcal mol^−1^ [33]. Laser ablation of graphite produces transient high-temperature plumes that permit controlled nucleation; it is the route by which the molecule was first identified [1] and by which that group first produced endohedral metallofullerenes in macroscopic quantities [34]. Fullerenes also form in fuel-rich hydrocarbon flames under restricted oxidation [35]. What all of these share is that they deliver isolated cages: they impose no control whatsoever over the topology of any network subsequently formed from them.

Within the classical routes, plasma methods deserve specific mention because they established that fullerene formation does not require graphite vaporisation at all. Xie et al. generated C_60_ in a microwave plasma struck in chloroform under a low-pressure argon atmosphere, extracting the products in toluene and separating them by liquid chromatography coupled to mass spectrometry; the same discharge produced four metastable C_60_ isomers alongside the Ih cage, which convert to buckminsterfullerene on heating and which constrain the cluster-closure mechanism [36]. Hetzel et al. converted graphite powder to C_60_ and C_70_ by microwave synthesis, identifying the products by MALDI-TOF mass spectrometry and emphasising the cost advantage of the approach over resistive heating [37]. Churilov and co-workers examined arc-discharge plasmas systematically and showed how strongly the yield depends on the discharge conditions: in a high-frequency carbon–helium arc the extractable fullerene content peaked at 12.6 wt% at 0.12 MPa and fell to around 7 wt% at higher pressures, while the proportion of C_70_ and higher fullerenes rose with pressure [38].

Precursor-directed molecular synthesis takes a very different approach. Surface-assisted cyclodehydrogenation and flash vacuum pyrolysis convert a designed, highly curved polyaromatic precursor into C_60_ through a defined sequence of ring closures rather than through statistical cluster growth. Yields are small and the routes are not competitive for bulk supply, but they are mechanistically informative in a way the plasma routes are not, because the product is determined by the precursor, not by the quench. The contrast is worth holding onto, since it recurs one level up: the topology of a 2D network is likewise fixed either statistically, by the conditions of polymerisation, or deterministically, by a template.

The magnesium-templated route supplies exactly that control. Growing layered polymeric Mg_4_C_60_ by chemical vapour transport and then removing the metal with dilute acid leaves a charge-neutral, purely carbon layered solid whose interlayer bonding can be weakened or cleaved while long-range in-plane order is preserved [3]. The topology of the resulting sheet is set during polymerisation rather than created during exfoliation. The original work established monolayer qHP and few-layer qTP C_60_, and the few-layer study that followed further demonstrated that covalent connectivity can persist in finite-thickness stacks [3,4]. Structural verification in these systems rests on a combination of techniques, not on any single one, since an apparently continuous nanosheet may still contain missing bridges, adsorbates, grain boundaries or other defects that dominate its response.

The 2025 electrochemical exfoliation route changed the practical synthesis landscape. Zhang et al. used in situ proton reduction to weaken interlayer interactions and promote tetra-n-butylammonium intercalation, reporting monolayer flakes of approximately 52.5 μm^2^, an exfoliation yield near 83% for that specific protocol, gram-scale throughput and direct integration into solution-processed photodetectors [39]. The importance of this result goes beyond the larger flakes; it links a scalable processing route to a device-level output. This yield figure should be read as a property of the protocol rather than of the material. It was obtained for in situ proton reduction combined with tetra-n-butylammonium intercalation of the Mg_4_C_60_-derived layered solid [39], under the particular electrolyte, potential and duration reported there. Exfoliation yields are in general not comparable between preparation methods. Reports differ in the denominator used, in the flake-size and thickness cut-offs applied before a sheet is counted as a monolayer, and in the technique used to assign thickness. A higher reported yield therefore does not by itself establish a more effective route.

Two 2026 developments further diversified processing. Yuan et al. reported potassium/sodium intercalation of Mg_4_C_60_ followed by benzonitrile quenching that released mono- and bilayer 2D-C_60_ sheets in less than ten minutes and produced approximately four-fold-higher photocurrent than bulk Mg_4_C_60_ [40]. Alotaibi et al. used high-shear flow in a vortex fluidic device to form covalently linked rectangular C_60_ sheets approximately 10–15 nm thick, about 20 μm long and 6 μm wide [41]. The latter material is not an atomically thin monolayer and is therefore best classified as a thicker 2D covalent C_60_ sheet, and not merged into the pristine-monolayer benchmark.

A complementary 2026 study expanded the experimentally accessible phase space, not the exfoliation route. Lv et al. synthesised hexagonal-phase (HP-2D-C_60_) and tetragonal-phase (TP-2D-C_60_) nanosheets and linked the narrower optical gap of the TP material to photothermal conversion in the second near-infrared window (NIR-II) [31]. This is direct evidence that tetragonal-phase 2D-C_60_ material can be prepared, but it does not by itself establish that the experimental TP topology is identical to qTP1, qTP2 or any other theoretical qTP model. The bond motif, layer number and molecular orientation remain the identifiers required for a quantitative comparison.

Coordination chemistry also provides a route for controlled deconstruction. Jia et al. showed that mild W- or Ni-coordination conditions can cleave inter-cage C–C σ bonds in robust two-dimensional fullerene frameworks and release intact C60 cages; removing Mg^2+^ markedly suppresses the depolymerisation, which indicates that intercalated Mg promotes junction opening under these coordination conditions [6]. The result complements phase-selective synthesis by demonstrating chemically triggered scission of the inter-cage bonds; because coordination and mechanistic modelling contribute differently to the evidence, the study is classified B/C at result level.

Characterisation of these materials is harder than for atomically flat crystals, and the difficulty is worth stating because it bears on how confidently structural claims can be made. Electron microscopy and diffraction establish long-range periodicity and the in-plane lattice parameters, and were the primary evidence in the original monolayer and few-layer reports [3,4]. What they establish less easily is the bridge motif itself, since the distinction between a single C–C link and a [2+2] bridge involves a small number of atoms at a specific site and is inferred from lattice metrics and simulation rather than imaged directly. Vibrational spectroscopy is the natural complement: the cage modes of C_60_ are well known, and their shifts and splittings on polymerisation report on the degree and symmetry of inter-cage bonding, which is how intercalation and demetallation steps are followed in practice [40].

Thickness determination deserves particular attention given the role it plays in Section 6.3. Atomic force microscopy returns an apparent height that includes the tip–surface interaction and any adsorbed layer, and for a corrugated cage-based sheet the relationship between that measurement and the quantity a simulation calls thickness is not one-to-one. This is not a criticism of the measurement but the reason the conversion problem exists: there is no experimental observable that uniquely fixes the thickness a calculation should divide by. Optical and near-field methods provide independent constraints, and combining several techniques is the most reliable route, since an apparently continuous sheet may contain vacancies, incompletely formed bridges, adsorbates or grain boundaries that dominate its response while remaining inconspicuous to any single probe.

A separate experimental line bears on how ordered cage assemblies form in the first place, even where no covalent network results. Bajoun Mbajoun et al. deposited C_60_ on the (100) surface of a complex Au-Si-Ho intermetallic and followed the growth by low-energy electron diffraction and scanning tunnelling microscopy: adsorption is disordered at room temperature but orders when the substrate is held near 669 K, with the molecules decorating pseudo-five-fold sites whose local geometry matches the cage, and the completed monolayer is structurally equivalent to a (100) plane of bulk fcc C_60_ [42]. The result is not a graphullerene, and it is retained here only as a benchmark under the scope rules of Section 2. Its relevance is the principle it isolates: molecular ordering can be dictated by geometric compatibility between substrate sites and cage facets, independently of any covalent chemistry. Since the phase obtained from a polymerisation route is now known to depend on the route, a templating step that fixes cage registry before bonding is one of the few plausible ways to select topology deliberately rather than statistically.

Processing has diversified faster than characterisation. Alongside chemical vapour transport with demetallation, electrochemical exfoliation and alkali-metal intercalation, mechanochemical routes using high-shear processing have been reported to assemble covalent sheets from C_60_ under ambient conditions [41]. The significance of this diversification is not that any one route is superior but that the phase obtained is now known to depend on the route, which converts synthesis from a preliminary step into a control variable. It also raises a question the field has not yet addressed systematically: whether sheets produced by different routes are structurally identical, or whether the defect populations and grain sizes differ enough that properties measured on one should not be transferred to another. Taken together, these developments changed what the field can attempt, and that is the significance of the synthesis effort rather than the arrival of one more carbon allotrope. Monolayer flake areas of tens of square micrometres with gram-scale throughput [39] move intrinsic monolayer measurement from impossible to merely difficult. That is what the comparisons in Section 6 and Section 10 ultimately lack: normalisation can remove the thickness artefact and a controlled cross-method benchmark can isolate the model dependence, but neither supplies a missing experimental value. Demetallation of an ordered crystalline precursor makes the connectivity reproducible and not only the composition, because the sheet topology is inherited rather than formed statistically [3]. The intercalation route of Yuan et al., which releases mono- and bilayer sheets in under ten minutes [40], makes layer-number series accessible, and layer number is the variable the thermal and electronic results require. The coordination-triggered cleavage reported by Jia et al. [6] shows that the network is chemically addressable rather than merely durable, which matters for post-synthetic modification and for recovery of the molecular precursor. Cumulatively, these advances provide an experimental platform on which structure–property relations can be tested one variable at a time. Figure 2 places these milestones on a timeline, and Table 2 records what each study established together with its evidence class.

## 5. Stability: Four Meanings That Must Not Be Conflated

The word ‘stable’ is used in at least four distinct senses in this literature: thermodynamic stability relative to competing phases; dynamical stability from phonon spectra; mechanical stability from the elastic tensor/Born criteria; and kinetic persistence over the duration and conditions of an experiment or molecular-dynamics trajectory. These criteria need not rank structures in the same order.

Peng’s analysis is the clearest demonstration. Within that structural set, qHP is thermodynamically less favourable than the competing monolayer phases over the temperatures considered, yet it is dynamically and mechanically more robust under strain [13]. One qTP variant has an extremely soft direction and a negative shear elastic constant, so an attractive negative Poisson ratio in the same harmonic description cannot be interpreted as a useful equilibrium auxetic property without stating the stability caveat. Conversely, Yan et al. later found mechanically and dynamically stable qTP-like and other phases in a different five-network structural set [14]. This is why the topology must be specified and not assumed to follow one universal qTP structure.

The practical lesson is that the four criteria answer four different questions and are not interchangeable. Thermodynamic stability answers whether a phase is the preferred product at equilibrium, which bears on an equilibrium-controlled synthesis, and on the long-term persistence of any phase once formed, but not on the immediate product of a kinetically controlled route—and the polymerisation routes used here are not equilibrium-controlled. The criterion is also defined only relative to a declared comparison. Thermodynamic stability is a statement about a phase with respect to a chosen reference set and a chosen set of computational conditions: the exchange–correlation functional, the dispersion treatment, whether vibrational entropy and finite temperature are included, and the pressure. A phase that is the least favourable member of one structural set can serve as the reference phase of another. That is why the energy ordering reported within Peng’s phase set [13] and that of the later five-phase survey [14] should not be read as a single ranking, and why a phase called less stable in one study is not thereby less stable than a phase called stable in another. Dynamic stability, read from the absence of imaginary phonon modes, answers whether a structure sits at a local minimum, which is a necessary condition within the harmonic, zero-temperature model in which it is evaluated, although anharmonicity, finite temperature, pressure or a substrate can stabilise a structure that the harmonic calculation rejects, and which says nothing about whether the structure can be reached. Mechanical stability, read from the Born criteria, answers whether the lattice resists arbitrary small strains, and a phase failing it will distort spontaneously regardless of its energy. Kinetic accessibility answers whether a synthesis pathway exists. A phase can satisfy any three and fail the fourth, and the experimentally realised phase does exactly that by sitting highest in energy among the candidates considered in that study.

This changes what a screening study can realistically tell us. Energy-ordered lists of candidate lattices are frequently presented as predictions of what might be synthesised, but if selection is kinetically controlled then position in such a list carries little predictive weight. A more useful output would pair each candidate with the bonding transformation required to reach it from an accessible precursor, since that is what determines whether a route exists. The magnesium-templated synthesis is instructive here: the phase obtained is set by the geometry the metal imposes on the precursor crystal before demetallation, not by the relative energies of the possible product sheets.

Thermal stability is similarly model-sensitive. Early reactive molecular dynamics (MD) work predicted destruction temperatures close to 3900 K for qHP/qTP [43]. A 2025 machine-learning-potential study using GAP-20 instead reported decomposition into individual C_60_ cages above roughly 1200 K [44]. These numbers should not be averaged. They reflect different potential-energy surfaces and definitions of structural failure, and they establish a validation problem, not a single melting or sublimation temperature. High-temperature claims therefore require cross-checking against ab initio data or experimentally constrained potentials.

Out-of-plane morphology adds another complication. The 2026 study of Zhao et al. predicts spontaneous wrinkling in suspended qHP sheets, size dependence and a dewrinkling–rewrinkling transition under tension [45]. This means that a nominally ‘flat’ in-plane tensile test can access morphology-dependent deformation modes before bond failure. Freestanding, supported and encapsulated systems must therefore be treated as different mechanical boundary-value problems.

Underlying all of this is the exceptional stability of the molecular building block itself. The isolated pentagon rule requires the twelve pentagonal faces of C_60_ to be separated by hexagons, avoiding the extra strain of adjacent pentagons [22]. C_60_ is the smallest fullerene satisfying that rule [22] and is produced efficiently by many high-temperature condensation routes [1,2]. The cage nevertheless enters the network already curvature-strained: closing an sp^2^ carbon surface onto a sphere pyramidalises the carbon sites, which contributes to the reactivity that permits inter-cage [2+2] bonding. The same residual reactivity that enables polymerisation can therefore also participate in thermally activated rearrangement or degradation.

Chemical degradability is orthogonal to the four stability criteria above. The coordination-triggered cleavage reported by Jia et al. does not imply that the pristine network is thermodynamically or mechanically unstable; it shows that an added coordination environment can redirect the reaction pathway and selectively lower the activation barrier for inter-cage σ-bond scission to values accessible under mild solution conditions [6]. Stability claims should therefore specify both the criterion and the chemical environment.

**Key stability insight.** The phase that is lowest in energy is not automatically the phase that can be isolated, remains phonon-stable, satisfies the Born criteria, or resists fracture. Any claim of “stability” should therefore state explicitly which criterion is being tested.

## 6. Mechanical Properties

### 6.1. Deformation Mechanisms and Anisotropy

The defining mechanical feature of covalent C_60_ sheets is not exceptional stiffness relative to graphene, but the way load is partitioned between rigid cages and softer inter-cage junctions. Under loading aligned with a dense bridge family, deformation is dominated by axial stretching of junction bonds and the response is comparatively stiff. Under transverse loading, cage rotation, bond-angle change, local flattening and junction rearrangement contribute more strongly, producing a softer and more non-linear response [18,21]. The elastic symmetry therefore follows the bond network more closely than an idealised hexagonal or tetragonal cage lattice.

The comparison that gives this statement its force is with the parent molecular solid. Nanoindentation measurements on fullerite and monomeric C_60_ films give Young’s moduli in the single-digit to low-teens GPa range [46,47], characteristic of solids whose inter-cage cohesion is dominated by van der Waals interactions. Covalently connected monolayers are substantially stiffer on the GPa scales reported in the literature, which is the central mechanical change introduced by polymerisation. Because the monolayer values depend on an effective-thickness convention, the quantitative comparison is developed more carefully on a two-dimensional basis in Section 6.3.

The individual measurements behind that range are worth naming, because the review literature tends to quote it without provenance. Nanoindentation of textured fullerite films condensed on KCl, LiF and Si returns a Young’s modulus of 14.1 GPa with a hardness of 0.42 GPa, in good agreement with the 14.7 GPa calculated for the (110) direction of the fcc lattice [46]; nanoindentation of monomeric C_60_ films gives 7.6 ± 3.6 GPa with a hardness of 0.3 ± 0.1 GPa, the lower value being the one that source reports as typical for monomeric C_60_ in the face-centred-cubic structure [47]. Both are properties of a solid whose inter-cage cohesion is dominated by van der Waals contacts, with at most a small covalent admixture, and the step to the covalent monolayers, a factor of three to thirty across the published pairs, is the quantity that justifies treating this family separately.

It is equally useful to bound the deformability of the isolated cage, since the cage sets what a junction can tolerate. Density-functional molecular dynamics at room temperature separates compression into three regimes [48]. The elastic range extends to a compressive strain of 0.48 ± 0.02 with only a weak orientation dependence; a force plateau near 30–32 nN then persists to about 0.75 ± 0.02 and contains stochastic bond ruptures; only beyond that does the deformation become irreversibly destructive. Two points matter for a network. First, the cage can accommodate substantial distortion before junction chemistry is necessarily lost. Second, bond rupture need not immediately destroy the cage, so local bond breaking at a bridge is not automatically equivalent to a propagating crack. This compliant, rotatable molecular unit is one reason the network response differs so strongly between loading directions.

First-principles studies provide the cleanest intrinsic elastic quantities. Shen et al. reported C_11_ = 140.2, C_22_ = 192.6, C_12_ = 23.9 and C_66_ = 63.5 N m^−1^ for a qHP monolayer, corresponding to strongly direction-dependent in-plane Young’s stiffness while satisfying the Born–Huang stability conditions [19]. Peng reported qHP values of 148.1/183.4 N m^−1^, qTP2 values of 146.4/145.2 N m^−1^ and the highly anisotropic qTP1 values of 5.4/123.5 N m^−1^ [13]. The qTP1 result is physically informative as a soft-mode signature, but it must be discussed together with the negative shear constant in the same model.

The full elastic tensor is more informative than a pair of Young’s moduli, and two components are especially important here. The off-diagonal constant governs the Poisson response, and the shear constant governs the resistance to in-plane distortion; the Born–Huang criteria require both, together with the diagonal constants, to satisfy inequalities without which the lattice is not mechanically stable at all. Applying those criteria to the candidate monolayers produces one of the more consequential results in the field: the quasi-tetragonal variant that exhibits the most extreme reported anisotropy, a ratio of more than twenty between its two principal directions together with a strongly negative Poisson ratio, has a negative shear constant and therefore fails the criteria [13]. Its auxeticity is a signature of instability, not an exploitable property, and it should not be quoted as a design target.

This matters directly for how anisotropy is reported. A single pair of orthogonal moduli characterises the response only along two special directions; for a lattice whose stiffness varies continuously with orientation, the complete description is the directional modulus as a function of angle, and stiffness and strength are not extremal in the same directions: in the one network for which the full angular dependence has been computed, the modulus is largest at 0° and smallest at 90°, i.e., on the crystallographic axes, while the ultimate tensile strength and the work to fracture are largest at 15° and smallest at 75° [21]. A pair of orthogonal moduli therefore locates the stiffness extrema correctly and still says nothing about where the strength extrema lie. Reporting the tensor, from which the polar plot follows, removes this ambiguity entirely and costs nothing, since the tensor is computed en route to the moduli in any case.

### 6.2. Strength, Fracture, Temperature and Defects

Fracture studies reveal greater method and temperature sensitivity than small-strain elasticity. Ribeiro Junior et al. used the ReaxFF reactive force field and reported qHP and qTP elastic moduli of 175.9 and 100.7 GPa, respectively, together with a fracture description that its abstract and its results section state differently [43]. Ying et al. reached a related conclusion by combining density functional theory (DFT) with a neuroevolution machine-learning potential and attributed the fragility of qHP to inhomogeneous deformation between rigid intra-cage bonds and softer inter-cage links [18]. Their finite-temperature MD results also showed that strength and failure strain can be far below ground-state DFT values.

The difference in fracture mode is at least as informative as the difference in modulus. The quasi-hexagonal network fractures along well-defined bonding pathways with a sharp, brittle load drop, and its crack trajectory is direction-dependent: linear under stretching along x and non-linear along y. For the quasi-tetragonal network the same paper is inconsistent with itself. Its results section reports that the lattice breaks linearly under stretching along both axes, by the same mechanism in each, while its abstract and conclusions describe the crack propagation as non-linear; we report that discrepancy rather than resolve it [43]. The linear trajectory of the quasi-hexagonal network is itself direction-dependent in the underlying data, linear under stretching along x and non-linear along y. A non-linear pathway may imply altered crack-tip stress redistribution and greater energy dissipation, but it does not by itself establish greater damage tolerance. That claim requires fracture energy or toughness, flaw-sensitivity and repeated-trajectory measurements under matched conditions. The available result therefore identifies a topology-dependent fracture pathway rather than a demonstrated reversal of application-level mechanical ranking.

Defects have received markedly less attention than ideal lattices, and the asymmetry matters because the two quantities of interest respond differently to them. The elastic modulus is a volume-averaged property of the bonding and is comparatively insensitive to a dilute defect population, so a defect-free calculation is expected to remain representative of it. Strength is not: it is governed by the largest flaw, so the intrinsic strengths computed for perfect sheets are upper bounds whose distance from a laboratory value is presently unknown. Missing cages, miscoordinated or absent bridges, Stone–Wales-type rotations and grain boundaries between domains of different orientation are all expected in material grown from multiple nucleation sites, and each acts as a stress concentrator. A systematic defect taxonomy linking specific imperfections to specific property changes does not yet exist for this family, and it is among the most tractable gaps identified in this review.

The defect problem also connects naturally to the wider graphene literature. The taxonomy of point and line defects in sp^2^ carbon—vacancies, Stone–Wales bond rotations, grain boundaries between misoriented domains—is well established for graphene, together with the general finding that such imperfections can be exploited to tailor local properties instead of only degrading them [49]. What does not transfer directly is the energetics: in a cluster-based lattice a defect may sit within a cage, within a bridge, or at the junction between them, and these are three distinct classes of imperfection with no graphene analogue. Adapting the existing framework rather than rebuilding it is the obvious route to the missing taxonomy.

A related methodological caveat concerns the classical potentials themselves. Comparisons across the AIREBO (adaptive intermolecular reactive empirical bond order), REBO and Tersoff families, and separately between ReaxFF and AIREBO-M, show that the computed response depends strongly on the potential: not only the absolute moduli and strengths but also the ordering of the strengths itself is not preserved between potentials [50,51], and the reason is specific to these materials: the transferability of an empirical carbon potential is established for planar sp^2^ and tetrahedral sp^3^ environments, whereas the inter-cage bridge is neither. Machine-learned potentials remove much of this arbitrariness but replace it with a dependence on training-set coverage, functional choice and dispersion treatment, and small energetic differences can reorder the stability of competing phases. This leads to the same conclusion as the thickness analysis: the potential is part of the reported result, and cross-study comparisons are meaningful only within compatible model families.

Temperature adds another layer of sensitivity in these cluster-based lattices. The reported collapse of strength between the ground state and room temperature is substantially larger than in graphene or hexagonal boron nitride [52]. A plausible interpretation is enhanced sampling of cage librations and junction distortions—internal soft modes absent from a rigid planar lattice. These modes can preload local bond geometries into less favourable configurations before rupture, but their contribution has not yet been isolated from the potential, strain-rate and size effects. Assessment for service above ambient temperature must therefore rest on finite-temperature simulation or experiment rather than extrapolation from 0 K elastic constants.

Reported destruction and melting temperatures need the same caution as strength values. Early reactive-molecular-dynamics work placed the thermal limit of these networks high, but subsequent machine-learned-potential studies find lower and model-dependent values [44], and the spread reflects how each model describes the bridge bonds whose rupture initiates decomposition. Because failure in a cluster-based lattice begins at a small number of junction atoms rather than uniformly across the sheet, a potential that slightly over-binds those specific configurations will substantially overestimate the survival temperature. Any quoted thermal limit should therefore be accompanied by the potential used, exactly as a stiffness should be accompanied by its thickness convention.

Mortazavi and Zhuang quantified this temperature effect with a machine-learning potential: for their qHP model the ultimate tensile strengths were approximately 7.0/8.8 GPa along the two principal directions near 1 K and about 3.3/4.2 GPa at 300 K, while failure strain fell from around 0.07 to about 0.029 [52]. Similarly, Ajori et al. investigated temperature, sample size, defect density and several classical potentials, reporting reductions in stiffness/strength/toughness with defects and strong temperature effects on failure metrics [50]. These studies collectively show that a single room-temperature tensile value cannot be inferred from 0 K DFT elasticity.

Substrate support and encapsulation change the problem again. Yu et al. reported effective moduli of 191.6 GPa for freestanding qHP and 531.4 GPa for a graphene-encapsulated qHP composite, with analogous increases for qTP [12]. The encapsulated value is a composite structural response and must not be listed beside intrinsic monolayer stiffness as though it were a property of C_60_ alone.

The magnitude of that environmental effect deserves emphasis because it exceeds every intrinsic difference discussed so far. Encapsulating a monolayer between graphene sheets raises the reported effective modulus from 191.6 to 531.4 GPa for qHP and from 134.7 to 504.1 GPa for qTP, factors of roughly 2.8 and 3.7 [12]. The reported mechanism is geometric: adding graphene shifts the positions of stress concentration away from those of the bare monolayer, and the supported and encapsulated structures reach a higher fracture strain. The same study reports that defects in the C_60_ layer then have almost no effect on the composite response, which matters for devices: the flaw sensitivity that limits a freestanding sheet is largely removed by encapsulation. These numbers are properties of a composite stack, however, and should never be entered into a comparison of intrinsic monolayer stiffness, which is why Table 3 marks them as not comparable, a stronger statement than unrecoverable.

### 6.3. Cross-Study Normalisation of Stiffness

A major avoidable source of disagreement is the conversion of an intrinsically two-dimensional elastic response into GPa. A 2D calculation determines an areal elastic constant or force per unit width. If a study reports a three-dimensional-looking Young’s modulus, the relation is Y_2D_ = Y3D × t, where t is the effective sheet thickness chosen by the authors. Because t is conventional, not uniquely observable, for a monolayer, two papers can report different GPa values for the same underlying sheet response.

In the published C_60_-network literature, declared thicknesses range over nearly a factor of two. Just as importantly, several highly cited GPa-based studies do not disclose enough information to recover the conversion. The correct response is not to guess their thickness, but to mark those entries ‘not recoverable’ and exclude them from a normalised meta-comparison.

The conventions actually adopted can be recovered from the primary publications, and they differ by almost a factor of two: 6.06 Å for the C_36_ network [11], 8.785 Å for the qHP layer thickness used in the comparative thermal-conductivity study [53], 8.8 Å for the porous C_60_ lattices [15], 10.4 and 10.6 Å for the qTP and qHP monolayers in the reactive-force-field study [51], and 12.2 Å taken from an atomic-force-microscopy measurement [52]. A study adopting 6.06 Å will report a stiffness roughly twice that of a study adopting 12.2 Å for the same underlying sheet response, before any physics enters. Listing these values matters, because the claim that unit convention contributes substantially to the apparent scatter is only as strong as the spread in the conventions themselves. Figure 3 shows the comparison that results once the declared thickness is divided out.

This normalisation changes the interpretation of the literature. The qHP Young’s moduli reported directly in GPa span approximately 49.6–218.5 GPa, a factor of about 4.4 when all principal-direction values listed in Table 3 are retained. The thickness-independent values that can actually be compared occupy a much narrower interval, approximately 93–189 N m^−1^ for the recoverable/native qHP entries in the same table. The residual spread is real and should be attributed to structural definition, force field, temperature, finite size, axis convention and elastic-window selection, and not to thickness convention alone.

The reduction can be quantified, but the two populations should not be treated as identical datasets. The raw GPa literature includes studies whose thickness is unstated and therefore cannot be normalised, whereas the thickness-independent set includes native 2D DFT results that were never expressed in GPa. On a simple min-to-max basis, the first spans about 4.4-fold and the recoverable/native 2D set about 2.0-fold. This is therefore an illustrative cross-study diagnostic, not a formal meta-analytic effect size. Its robust conclusion is qualitative but important: removing recoverable thickness conventions narrows the apparent mechanical range, while the remaining differences must be traced to topology, temperature and modelling protocol.

Normalisation does more than tidy the bookkeeping; it changes the interpretation of individual stiffness claims. The C_36_ network, for example, was reported at 266 GPa using a 6.06 Å thickness [11]. On the corresponding two-dimensional basis this is about 161 N m^−1^, which lies within the qHP first-principles range, not above it. The GPa value therefore should not be used to claim a larger intrinsic 2D stiffness than qHP. Porous C_60_ lattices provide the opposite example: values approaching 300 GPa with an 8.8 Å convention correspond to about 264 N m^−1^ [15], which remains above the dense qHP values after the same conversion. That result supports the more defensible conclusion that bond orientation and load path, not atomic density alone, can dominate stiffness in this family.

The primary reactive-molecular-dynamics data of Ribeiro Junior et al. also illustrate why both direction and axis definition must be retained [43]. The study reports 175.9 GPa along x and 218.5 GPa along y for qHP, together with 100.7 and 133.5 GPa for qTP. Using only one value per phase hides the anisotropy and can create an artificial discrepancy with other studies. In the coordinate system used by Ribeiro Junior et al., qHP is stiffer along y, consistent in sense with Peng’s first-principles qHP result [13] but opposite to the x/y ordering reported in the targeted reactive-force-field study [51]. Because the underlying structural axes are not named consistently across these papers, this difference suggests an axis-mapping problem and should be treated as one until the bridge orientations are explicitly aligned, and not as evidence that the material reverses its anisotropy.

The force-field comparison can also be stated more precisely. Giannopoulos et al. held the geometry, code and protocol broadly fixed while changing between their tested ReaxFF and AIREBO-M descriptions [51]. For the qHP Young’s modulus, the difference is around 6% in each principal direction. This does not justify a universal statement that ‘force fields differ by only 6%’; it establishes a controlled same-protocol result for one observable and one model pair. However, strength, fracture energy and high-temperature stability can be substantially more sensitive.

**Reporting rule 2.** For monolayers, report C_ij_ and Young’s stiffness natively in N m^−1^. If GPa is also supplied, state the exact effective thickness and give the formula used. Never compare a composite encapsulated modulus with an intrinsic freestanding modulus without an explicit structural normalisation.

The record of what has actually been reported is collected in Table 4, which is deliberately inclusive: it carries the molecular and bulk baselines, the first-principles elastic results, the reactive and machine-learned molecular-dynamics values, and the substrate-supported composites in a single place, in the units each study used. It is a record, not a ranking, and the entries should be read against the normalisation of Section 6.3 and the evidence classes of Section 2.2 before any two rows are compared directly.

## 7. Tribology and Interfacial Sliding

Interlayer friction is the property in which the cage-based surface departs most sharply from flat sp^2^ carbon, because the corrugation of the sliding potential is set by the cages, not by individual atoms. Several published interface studies reach conclusions that are not obviously reconcilable at first sight, and that disagreement is informative. Structural superlubricity is reported for clean, incommensurate interlayer contact, where the sliding barriers are largely cancelled and the friction collapses to the very low values characteristic of that regime [55], a picture extended to graphullerene-based van der Waals heterostructures [56]. In contrast, simulations under finite load and sliding velocity find a pronounced stick-slip response with the friction force rising strongly with velocity, together with puckering ahead of the slider and, at high velocity, intermittent loss of close contact [57].

These results are better interpreted as two regimes of the same system rather than as a contradiction, since superlubricity is a property of the clean, lightly loaded, incommensurate contact while stick-slip and puckering appear under load and at finite velocity. The boundary between them has not yet been mapped, which makes it a clear target for future work. The application-level assessment is less favourable and more instructive: compared directly against graphene as a lubricant additive, the mixed sp^2^/sp^3^ bonding lowers the in-plane strength while the closed cages raise the bending stiffness, and under boundary lubrication graphene outperforms graphullerene because that same bending stiffness prevents the conformal deformation that would relieve contact stress [58]. Under fluid lubrication the rigidity suppresses the low-shear solid–liquid interfacial slip that graphene promotes, forcing higher-resistance liquid–liquid slip instead.

The same lesson extends beyond tribology: an intrinsic property measured on an idealised interface does not transfer directly into application performance, and the same structural feature can be responsible for both outcomes. The rigidity conferred by the closed cage flattens the sliding energy landscape in the ideal contact and simultaneously removes the conformability a real lubricating film requires. Reporting either result without the other would misrepresent the material.

The tribological results also connect back to structure in a way that is specific to this family. The corrugation of the sliding potential is set by the cage spacing, not by the C–C bond length, so its period is roughly an order of magnitude longer than in graphene. A slider therefore samples a smoother but longer-wavelength landscape, which favours superlubric cancellation for incommensurate contacts while making the response more sensitive to flake size and to the relative rotation between surfaces than in atomically corrugated systems [55,56]. This suggests that the tribological behaviour of these networks should be reported as a function of contact area and twist angle rather than as a single friction coefficient, and that comparisons between studies using different flake sizes are not meaningful without that information.

Qiu et al. combined indentation, tensile and friction simulations and emphasised that the mixed sp^2^/sp^3^-like junction environment and high bending stiffness alter contact mechanics relative to graphene [58]. In 2026, however, Yang et al. extended the heterostructure concept to graphullerene/graphene, graphullerene/MoS_2_ and graphullerene/h-BN and found robust superlubricity over broad ranges of twist, load, velocity, direction and temperature, with finite-size edge pinning remaining comparatively weak [56]. Likewise, Zhao, Guo and Qiu showed in molecular dynamics that a monolayer polymeric C_60_ nanoflake can undergo persistent one-dimensional Brownian motion on unpatterned graphene, h-BN and MoS_2_ surfaces because the symmetry-broken interfacial energy landscape supplies intrinsic directional tracks [59]. These studies extend the interface problem beyond a single friction coefficient toward controlled translation, registry and nanoscale transport.

## 8. Electronic and Charge-Transport Properties

Covalent linking converts molecular orbitals of isolated C_60_ into directionally coupled bands while retaining substantial molecular character. The experimentally realised qHP network is semiconducting and anisotropic [3]. However, the phrase ‘the graphullerene band gap is 1.6 eV’ is too coarse for a critical review. The numerical value depends on both structure and observable: experimental transport gaps, semilocal Kohn–Sham gaps, hybrid-functional gaps, quasiparticle GW gaps and optical onsets are not interchangeable.

Yu et al. predicted a direct gap of about 1.55 eV for an experimentally motivated monolayer model and linked the electronic anisotropy to asymmetric bridge arrangements [17]. Li and Sun subsequently compared bulk and monolayer qHP/qTP structures and found that the bridge bonds carry a large share of the local electron density while the optical response remains strongly structure- and polarisation-dependent, particularly for monolayer qTP models [60]. Peng used semilocal and hybrid-functional treatments in the context of water splitting and obtained phase-dependent gaps in the ~1.7–1.9 eV range with the weakly screened hybrid approach [61]. In contrast, Yan et al. later found semiconductor gaps between 0.85 and 1.55 eV across five distinct C_60_ network patterns [14]. These values demonstrate structural and methodological tunability, not a single inconsistent measurement of one universal gap.

The physical origin of the semiconducting response of experimentally anchored qHP is worth making explicit. In graphene the pi system is delocalised over an infinite planar lattice and the valence and conduction bands touch at the Dirac point. In molecular C_60_ the frontier orbitals are discrete, while the molecular solid is coupled mainly by van der Waals interactions. A covalent qHP network sits between these limits: inter-cage bridges broaden the molecular levels into dispersive bands while preserving substantial cage character. The resulting gap is therefore inherited from the molecular electronic structure and modulated by connectivity, strain and stacking. This picture should not be generalised to every hypothetical fullerene network, because broader topology screening also predicts metallic and semimetallic C_60_ lattices [15].

Stacking supplies a further degree of freedom that has no monolayer analogue. Bilayer assembly modifies both the gap and the optical response through interlayer coupling [62], and in trilayer quasi-hexagonal stacks a sliding-induced ferroelectric polarisation has been predicted, arising from symmetry breaking and interlayer charge redistribution, not from ionic displacement [63]. A dielectric substrate adds another control variable: DFT modelling of C_60_-polymer/h-BN bilayers found stable van der Waals heterostructures with modified electronic and optical response relative to the isolated polymer [64]. Dimensional reduction provides a boundary case, not part of the 2D core: polymeric C_60_ chains can improve calculated photocatalytic driving forces [65], while nanoribbons cut from qHP/qTP networks develop width- and edge-dependent direct/indirect gaps and qHP edge states [66]. Overall, the electronic structure cannot be reduced to a single fixed material constant; it depends jointly on bridge topology, layer number, registry, strain, environment and chemical modification.

### 8.1. Mobility and Polaronic Transport

Two independent modelling approaches now place polaronic physics at the centre of charge transport in qHP C_60_. Cassiano et al. used a Holstein–Peierls Hamiltonian and obtained stable large polarons as the primary carriers, with an anisotropy coefficient of at least 50% and field-driven velocities of roughly 0.5–1.5 nm ps^−1^ [67]. Similarly, Capobianco et al., using a quantum-mechanical electron-transfer treatment, found polaronic localisation energies of roughly 0.1 eV in both molecular fullerite and qHP graphullerene; in their picture, the higher qHP mobility arises from stronger inter-cage electronic coupling, not from elimination of localisation [68]. The methods are different, but both results argue against treating a simple effective-mass band mobility as a direct prediction of transport in a real sheet.

The distinction is familiar in molecular semiconductors but is easily lost when the material is described as a two-dimensional crystal. Band transport assumes carriers delocalised over many unit cells with scattering as a perturbation; polaronic or hopping transport allows substantial carrier-lattice localisation and thermally or field-assisted motion. Cluster-based lattices sit between these limits because intra-cage bonding is strongly delocalising while inter-cage coupling is weaker and geometry-sensitive [67,68]. Mobilities derived from band effective masses and mobilities derived from hopping or polaron models are therefore different observables and cannot be pooled into a single comparison without stating the transport model.

### 8.2. External-Field, Strain, Stacking and Impurity Control

Electric field and stress provide additional tuning dimensions. Miao et al. predicted field-dependent shifts in the qHP/qTP band structure and conditions under which semiconductor-to-conducting behaviour emerges [69]. Wang et al. showed that applied stress changes the anisotropic electronic and optical response [70]. Bilayer stacking leaves the band gap and band-edge alignment broadly similar to the monolayer in the study by Shearsby et al., while enhancing the anisotropy of carrier effective masses and strengthening visible excitonic absorption [62]. Trilayer qHP has also been predicted to exhibit cluster-sliding ferroelectricity, including switchable in-plane and out-of-plane polarisation states [63].

Impurity engineering expands the design space further, but the structural model must be stated because qHP and qTP doping studies are not interchangeable. Yadav examined BN co-doping of a qTP2-type polymeric C_60_ sheet and found that substitution changes the band gap, optical response and deformation-potential transport while preserving a semiconducting state over the configurations considered [24]. Yadav, Sadhukhan and Yadav subsequently studied BN-doped qHP C_60_ with first-principles calculations and classical simulations, again finding strong electronic and optical sensitivity to dopant concentration [25]. Khan et al. pursued a different route, predicting spin-dependent donor/acceptor states from B, N and H impurities with magnetic moments close to 1 μ_B_ per impurity [26]. These are class-D functionality predictions: they show how the electronic structure may be engineered, but they should not be described as experimentally demonstrated doping or spintronics. Table 5 is included as a reading instrument rather than as a summary of results. Its function is to prevent one specific error, the comparison of two numbers that are not the same observable. Each row therefore binds a reported quantity to the method and the structural model that produced it, and the final column states the conclusion that value can and cannot support. Rows are intended to be read across rather than ranked down the column.

Read together, the rows of Table 5 support three conclusions and refuse a fourth. The quasi-hexagonal monolayer is the one phase whose structure is anchored experimentally, and it is computed to be semiconducting with a gap in the visible range and a pronounced in-plane anisotropy of the carrier effective masses [62]—the structure is experimental while these electronic properties are not. The reported gap values differ in the first instance because they are not all the same observable, and because the table is a heterogeneous collection rather than a controlled comparison, the residual method dependence cannot be apportioned from it. The transport description is contested at the level of mechanism, with band-like and polaronic pictures both defended for the same material by Cassiano et al. and Capobianco et al. [67,68]. What the table does not support is a single quoted mobility for this family: the reported values belong to different transport regimes, temperatures and structural models, and no four-terminal monolayer measurement with contact-resistance correction exists against which to select among them.

Endohedral encapsulation offers a different perturbation because the carbon network can remain intact while the electronic states inside the cages are changed. Pereira Junior et al. investigated N, Ce and Sr encapsulation in qHP C_60_ at several filling fractions using PBE-DFT [27]. Nitrogen preserved the semiconducting character while introducing localised intragap states; Ce and Sr produced metallic behaviour at full and intermediate encapsulation, with semiconducting behaviour re-emerging at the lowest filling considered. The optical onset was correspondingly red-shifted. These results enlarge the design space, but they remain class-D predictions until endohedral qHP networks are experimentally realised.

A precedent from a different material system indicates why this design frame deserves attention, rather than treating fields and impurity states only as degradation channels. In CMOS-integrated silicon p + n junction diodes operated in avalanche breakdown, carriers heated by the junction field produce electroluminescence at photon energies far above the indirect gap of silicon, with junction defect states implicated in the emission, which converts a notoriously inefficient emitter into a monolithically integrated on-chip optical source, as Chen et al. demonstrated [73]. For C_60_ networks the corresponding question, whether field-driven carrier heating combined with deliberately introduced impurity states can deliver a function the pristine network does not possess, has not been examined: the doping and encapsulation studies above stop at the equilibrium electronic structure, and none of them considers a high-field or hot-carrier operating regime. None of the studies reviewed here addresses it.

### 8.3. Magnetic Response and the Molecular Baseline

Magnetism in this family is almost entirely a property of what is done to the cages, not of the cages themselves, and the molecular baseline makes that explicit. Pristine C_60_ has a closed-shell configuration and is diamagnetic. A paramagnetic response appears only when that shell is opened, which occurs by charge transfer from a donor, by radical formation on the cage surface, by structural defects that leave unpaired spin density, or by encapsulation of a magnetic species inside the cage. The molecular solid therefore offers a set of well-characterised routes from a diamagnetic host to a spin-carrying material, none of which requires a magnetic element in the lattice.

The most consequential of these is alkali-metal doping. Charge transfer into the three-fold-degenerate LUMO of C_60_ converts insulating fullerite into a metal, and at the A_3_C_60_ stoichiometry the material superconducts, the first report being potassium-doped C_60_ at 18 K [74]. The mechanism is understood to involve strong coupling between the carriers and intramolecular vibrations, which is to say that the molecular character retained by the solid is not incidental to the effect but responsible for it. This is the clearest historical demonstration that the electronic ground state of a fullerene solid is a controllable variable, not a fixed property, and it is the precedent against which doping studies of the covalent networks should be read.

What has been established for the 2D networks is considerably narrower, and the distinction should be maintained. No experimental magnetic ordering has been reported in a covalent C_60_ monolayer. Khan et al. predicted that B, N and H impurities generate spin-polarised donor/acceptor states with moments close to 1 μ_B_ per impurity [26], which is a half-semiconducting functionality prediction, not a demonstrated spintronic material. A complementary calculation by Suo et al. placed Mn, Fe, Co and Ni single atoms on qHP C_60_ and predicted low-barrier O_2_ activation and CO oxidation; in their interpretation the network acts not only as an electron reservoir but also as a ‘spin sponge’ that accommodates the reduction in the O_2_ spin moment [71]. These studies make magnetically active chemistry plausible on the network. However, neither establishes long-range magnetic order in a pristine sheet.

## 9. Optical and Excitonic Properties

The optical response is particularly sensitive to the quasi-molecular character of the network. Standard DFT can describe qualitative band anisotropy but does not capture the strongly bound electron–hole pairs expected in a weakly screened quasi-2D material. Champagne et al. used GW-BSE and found exciton binding energies up to about 0.8 eV in monolayer graphullerene together with strong linear-polarisation anisotropy [75]. Residual or incorporated Mg changes both structure and screening, renormalising the quasiparticle gap by roughly 1 eV and reducing the calculated exciton binding to about 0.6 eV [75].

The molecular fullerene picture provides a useful starting point. Isolated C_60_ absorbs across the ultraviolet and, weakly, into the visible through pi-pi* transitions of the closed cage, with a HOMO-LUMO separation of roughly 1.6–1.9 eV; the lowest transitions are symmetry-forbidden, which is why the molecule is a poor absorber in the visible despite its extended conjugation. Its defining photophysical property is what happens next: intersystem crossing to the triplet manifold is close to quantitative, the triplet is long-lived, and it transfers energy efficiently to molecular oxygen to generate singlet oxygen [76]. That combination—near-unit triplet yield, high electron affinity, long-lived excited state—is the basis of essentially every photochemical application of molecular fullerenes.

How much of it survives polymerisation is an open and consequential question. Covalent bridging consumes cage carbon atoms, broadens the discrete levels into bands and introduces the strong dielectric screening effects discussed above, all of which act against the sharply defined, weakly coupled excited states that the molecular photophysics depends on. The excitonic picture that replaces it is different in kind, not a perturbation of the molecular one. Where a network is proposed for a photochemical function, therefore, the molecular precedent establishes plausibility but not performance, and the relevant quantities—triplet yield, singlet-oxygen quantum yield, excited-state lifetime—have not been measured for any covalent C_60_ sheet.

Optical anisotropy has now been reproduced in several independent calculations. Li and Sun reported strong visible/near-infrared absorption and pronounced polarisation dependence for monolayer qTP models [60]; Wei et al. analysed the response as a function of cage orientation and reinforced the view that polarisation is a structural fingerprint of the inter-cage network [77]; and Shearsby et al. found stronger visible exciton absorption and enhanced polarisation dependence in bilayers [62]. Xu et al. extended the question from static spectra to real-time excited-state dynamics using velocity-gauge density-functional tight binding: water markedly lengthened electron–hole recombination in molecular C_60_ but had comparatively little effect on qHP C_60_ [72]. This result suggests that the covalent network can retain its excited-state dynamics in a solvated environment. Consequently, optical comparisons should report not only the many-body level of theory and layer count but also the dielectric/solvation environment when time-resolved or excitonic quantities are discussed.

A methodological caution applies to excitonic quantities specifically. Unlike a Kohn–Sham gap, the quasiparticle gap and exciton binding energy are sensitive to physical dielectric screening from substrates, encapsulation or solvent and to numerical convergence in a periodic slab calculation. Vacuum spacing is a convergence parameter rather than a physical dielectric environment: without Coulomb truncation, periodic-image interactions can contaminate the result, while a converged truncated calculation should minimise that artefact. Reported values are therefore comparable only when the physical environment, Coulomb treatment, k-point sampling and vacuum convergence are documented [75]. The qualitative conclusion—that these networks host strongly bound, anisotropic excitons because of weak screening and residual cage-derived localisation—is supported by independent calculations, while specific numerical values must remain attached to their computational setting.

These two strands, the optical response and the excitonic structure, are not separate results but two views of one quantity, and the relation between them is quantitative. Where the lowest exciton is optically allowed, the absorption onset of such a sheet is the quasiparticle gap reduced by the exciton binding energy. With the binding energies of up to about 0.8 eV obtained by Champagne et al. [75], the optical and quasiparticle gaps are offset by an amount comparable to the entire spread between exchange–correlation treatments. An optical onset and a computed gap are not two estimates of the same number, and a measured onset cannot be used to validate a computed gap unless the binding energy is supplied with it. Where the lowest exciton is dark, which the symmetry-forbidden lowest transitions of the molecular precursor make a real possibility here, the measured onset belongs to the lowest bright exciton and lies above that difference, so a computed binding energy taken from the lowest state is an upper bound on the offset rather than an estimate of it.

The polarisation anisotropy is likewise a property of the exciton and not of the band structure alone. Its transition dipole is carried by the directional inter-cage coupling, while the electron–hole interaction is screened anisotropically in the same lattice; whether those two anisotropies reinforce or partly offset one another has not been determined for this material. The two many-body corrections also do not cancel: incorporated Mg reduces the calculated exciton binding energy by only about 0.1 eV while renormalising the quasiparticle gap by about 1 eV [75], so the optical onset follows the quasiparticle renormalisation and the excitonic term modifies it by roughly a tenth of that amount. Any comparison across studies must consequently state which of the three gaps, Kohn–Sham, quasiparticle or optical, is being quoted.

The practical significance of the optical anisotropy is that it can make these networks intrinsically polarisation-sensitive without patterning or an external field. Absorption differs between the two principal in-plane directions because the inter-cage coupling that carries the transition dipole is itself directional, following the bridge motifs [17,60,77]. A device built on such a sheet could therefore discriminate polarisation as a material response rather than only through an engineered grating or metasurface. Combined with a gap in the visible range, this makes polarisation-resolved photodetection a natural application to test experimentally, although device-level polarisation selectivity has not yet been established.

Chemical modification provides a second handle on the optical response. Incorporating metal species during synthesis alters both the polarisability and the dielectric screening, which reduces the exciton binding energy and renormalises the quasiparticle gap; the effect is not a rigid shift in the spectrum but a change in the balance between single-particle and many-body contributions to it. Strain acts differently again, shifting absorption features by altering the band dispersion directly [70], and in the smaller-cage networks the same strain produces larger shifts because the bridges occupy a greater fraction of the structure. The optical behaviour of these materials is consequently not fixed by composition; it emerges from the interaction between molecular identity, network architecture and environment, and each of those can be adjusted independently.

**Reporting rule 3.** For electronic/optical studies, label the observable explicitly: Kohn–Sham gap, hybrid-functional gap, quasiparticle gap, transport gap, optical onset or exciton binding energy. A single “Eg” column without this distinction is not a valid cross-study benchmark.

## 10. Thermal Transport and Thermal Expansion

The starting point is the parent solid. In single-crystal C_60_ fullerite the thermal conductivity is about 0.4 W m^−1^ K^−1^ at room temperature [78], far below graphene and other high-conductivity extended sp^2^ carbon materials because the van der Waals coupling between neighbouring cages does not sustain fast propagating phonons. Vibrational energy is carried mainly by low-frequency inter-cage modes, while much of the high-frequency intra-cage spectrum remains comparatively localised. The result is a crystal with glass-like thermal transport despite long-range order, a useful reminder that crystallinity alone does not guarantee high thermal conductivity.

Compared with van der Waals fullerite, covalent polymerisation can increase in-plane thermal transport substantially by opening bonded pathways between cages [53,79]. The magnitude is not yet settled: credible monolayer simulations range from only a few to more than 100 W m^−1^ K^−1^, so no single order-of-magnitude value should be treated as intrinsic. Across the reported models, transport remains far below graphene and is strongly anisotropic, following the bridging directions. Stacking then produces an effect that runs against the intuition carried over from graphene: bilayer networks conduct less well than monolayers, because the second layer redshifts precisely the sub-10 THz branches that carry the heat, lowering their group velocity and mean free path, while stronger interlayer coupling intensifies the anharmonicity and localises those modes [79]. Interlayer registry is therefore an active scattering channel, not a passive stacking variable, and layer count must be reported alongside any conductivity value for this family.

Interfaces add a further layer. In graphene-C_60_ heterostructures the transport can be governed as much by the resistance at the junction as by the conductivity of either component, because the high-velocity planar modes of graphene couple imperfectly to the low-frequency cage-centred modes of the network [80]. A large fraction of vibrational energy can therefore be scattered at the boundary and cross-plane heat flow suppressed even when each layer retains substantial in-plane transport. The combination emerging from the literature—moderate-to-high in-plane stiffness together with much lower thermal transport than graphene in most reported cases—is unusual among carbon-based 2D materials and underpins the thermoelectric interest in these networks [81].

Thermal transport is the property for which the literature currently shows the largest unresolved quantitative spread. The approximately 2.7 W m^−1^ K^−1^ experimental value associated with the Meirzadeh et al. material was obtained by steady-state thermoreflectance on a few-micrometre-thick qHP C_60_ specimen, not on an isolated monolayer [4,82]. It should therefore be treated as an experimentally anchored layered-material baseline, and not as a monolayer measurement. For monolayer qHP, Mortazavi and Zhuang obtained about 2.9 and 5.7 W m^−1^ K^−1^ along the two principal directions at 300 K using a machine-learning potential [52].

However, a very different result was obtained by Dong et al. using a neuroevolution potential and homogeneous non-equilibrium MD: approximately 102 and 137 W m^−1^ K^−1^ along the two qHP principal directions at 300 K [53]. In contrast, Hu et al. more recently used a moment-tensor potential and found qHP values around 16.9 and 23.1 W m^−1^ K^−1^, with a wider family of C_60_ networks spanning roughly 9.2–34.4 W m^−1^ K^−1^ [82]. These studies cannot all be summarised as ‘κ ≈ 10 W m^−1^ K^−1^’. The disagreement is a major open benchmarking problem involving the potential, phonon lifetimes, cell size, thickness convention, structural mapping and transport algorithm. Figure 4 plots the reported values and makes the size of the disagreement visible at a glance.

Layer count introduces a separate effect. Yang et al. found that bilayer qHP/qTP networks conduct less efficiently in-plane than their monolayers because stacking redshifts and localises the low-frequency phonon branches that carry a large fraction of the heat [79]. Hu et al. later showed in 2025 that the number and shape of covalent inter-cage connections can systematically tune group velocities and lifetimes [82]. In 2026, overlapping-network simulations predicted a non-monotonic total thermal resistance with overlap length, while 5% vacancies reduced intrinsic conductivity by more than 78% [80].

Thermal expansion adds a structurally elegant result. Shaikh, Wu and Peng showed that nearly spherical molecular networks can exhibit opposite expansion signs along orthogonal bond families: positive expansion along [2+2] cycloaddition directions and negative expansion along intermolecular C–C single-bond directions [23]. This offers a direct structure-to-function design rule and illustrates why scalar thermal-expansion coefficients are inadequate for qHP-type lattices.

Thermal transport and thermal expansion are reported separately in this literature, but at a given temperature both respond to the anharmonicity of the same inter-cage bridges, and the connection is worth making explicit. Their shared ingredient is the cubic force constants of those bridges, which enter the two properties through different contractions: the expansion through the mode Grüneisen parameters weighted by the heat capacity, the intrinsic three-phonon lifetime through the scattering rates. This ingredient is not the whole of either property, and least of all of the conductivity, which depends additionally on the harmonic dispersions, group velocities and heat capacities, and in any real sample on four-phonon, boundary and defect scattering. The sign of the expansion therefore carries no direct implication for the conductivity along the same direction, and the opposite expansion signs of the two bond families [23] must not be read as a conductivity ordering.

What the shared origin does support is weaker. A direction along which the mode Grüneisen parameters are large in magnitude, whatever their sign, is a candidate for strong anharmonic scattering, but only a candidate: the scattering rate also depends on the full third-order matrix elements and on the available three-phonon phase space. A measured expansion tensor convolves those parameters with the full elastic compliance, so it constrains the anharmonicity of the two bridge families without isolating either of them, and any consequence for the conductivity has to be computed rather than inferred from it.

There is a practical consequence as well. A conductivity computed for 300 K on a cell relaxed at zero temperature omits the expansion altogether. Because that expansion is anisotropic and changes sign between the bond families, the resulting error has a magnitude component and a direction-dependent component, of which only the first could be absorbed into a scalar correction; it is a plausible contributor to the spread between Mortazavi and Zhuang, Hu et al. and Dong et al. collected in Table 6 [52,53,82]. No published study reports the conductivity and the expansion for the same structure, with the same interatomic model, at the same temperature, so the relation above remains a hypothesis rather than a result, and studies reporting either quantity should state the thermal state of the cell they used, as the reporting standard of Section 13 asks.

The breadth of the disagreement in reported conductivities deserves to be quantified rather than described. For nominally qHP monolayers, published simulations range from about 2.9–5.7 W m^−1^ K^−1^ [52], through approximately 16.9–23.1 W m^−1^ K^−1^ [82], to roughly 102–137 W m^−1^ K^−1^ [53]. This spread is larger than the directional anisotropy within any one of those studies and larger than many reported phase or layer-number effects. A structural conclusion drawn by comparing values across different transport algorithms and potentials is therefore not secure unless the model dependence is isolated. At present, thermal conductivity is the clearest property in this review for which the quantitative literature is method-limited, not experimentally anchored.

The thermoelectric interpretation therefore needs some caution. A low lattice thermal conductivity combined with a moderate gap and reasonable carrier mobility is the standard recipe for a thermoelectric material, and these networks have been proposed on exactly that basis [81]. However, a figure of merit computed from a conductivity uncertain by an order of magnitude is itself uncertain by the same factor, and the electronic contribution depends on the transport regime that Section 8.1 shows is unresolved. The qualitative case remains attractive and is supported by the physical mechanism, namely the spectral separation between inter-cage and intra-cage vibrations. The quantitative case cannot be settled until at least one direct measurement exists. Table 6 lists the representative values and the conclusion each supports.

**Thermal benchmark conclusion.** Thermal conductivity should currently be presented as a method-, direction-, topology- and layer-indexed range, and not as a single material constant. This remains the clearest property for which controlled cross-potential calculations and direct monolayer measurements are needed.

## 11. Applications: Evidence After the First Synthesis

Two kinds of evidence appear in this section, and they are labelled throughout rather than left to be inferred. A demonstration is a performance actually measured on material that was synthesised, and it is reported here together with the material it was measured on, whether monolayer, few-layer, or a supported or metal-decorated derivative. A prediction is a computed performance for a structure, dopant or active site that has not been realised experimentally, and it is reported as a prediction even where the computed figures are highly specific. The two are never combined into a single performance claim, the evidence classes of Section 2.2 are applied at result level, and the material-status column of the table of application-directed studies at the end of this section carries the same distinction for every entry in it.

### 11.1. Photocatalysis and Electrocatalysis

Theoretical work first identified monolayer C_60_ networks as promising photocatalysts because suitable semiconducting gaps, band-edge positions and exposed surface sites can coexist in a single carbon framework [61]. A related but distinct reaction was then demonstrated experimentally in 2023, when Wang et al. reported visible-light conversion of pure water by a few-layer fullerene network without sacrificial reagents, with H_2_ and H_2_O_2_ evolution rates of approximately 91 and 116 μmol g^−1^ h^−1^, respectively [84]. That reaction is two-electron water oxidation to hydrogen peroxide paired with hydrogen evolution, and it is not overall water splitting into H_2_ and O_2_; the distinction is maintained throughout this section. The predicted overall-water-splitting function is not merely undemonstrated on these networks: it was tested for experimentally and not found. In oxygen-18-labelled water, with synchrotron vacuum-ultraviolet photoionisation mass spectrometry, the same authors measured no labelled O_2_ among the gaseous products, assigned the residual O_2_ signal to air contamination, and confirmed H_2_ evolution independently by gas chromatography, concluding that the few-layer network has poor activity for the four-electron O_2_-evolving process [84]. The slight excess of H_2_O_2_ over H_2_ in the reported rates is attributed in the same work to reduction in a small amount of adsorbed O_2_, the main H_2_O_2_ pathway being the two-electron oxidation of water. What has been demonstrated, then, is simultaneous H_2_ and H_2_O_2_ production on a few-layer network. The distinction between theory and experiment is important: the monolayer first-principles prediction concerns ideal phases, whereas the demonstrated catalyst was a few-layer experimental network.

The catalytic literature has expanded from using the pristine network as a photocatalyst to using it as an electronically active support. Few-layer C_60_ nanosheets decorated with Bi nanoparticles achieved selective electrocatalytic CO_2_ reduction to formate [85], while ultrasmall Ir nanoparticles on monolayer covalent C_60_ gave a hydrogen evolution reaction (HER) overpotential of 18 mV at 10 mA cm^−2^ and long-term operation [29]. In 2025, three further experiments established a recurring ‘electron-buffer’ role for the covalent network: Pt nanoparticles on a polymeric C_60_ network reached a mass activity of 118 A gPt^−1^ for ammonia oxidation [86]; Ir nanoclusters on C_60_NET reached 237 mV OER overpotential with operation beyond 600 h at 10 mA cm^−2^ [87]; and Ru nanoparticles on few-layer covalent C_60_ required 24 mV at 10 mA cm^−2^ for alkaline HER and showed about eight-fold-higher intrinsic activity than unsupported Ru nanoparticles [88]. These results are not properties of pristine C_60_ alone, but they demonstrate experimentally that the network can participate electronically in metal-support catalysis rather than behaving as an inert scaffold.

The appeal of these networks for photocatalysis comes from a specific combination of requirements, not from one property alone. Overall water splitting, which is the reaction targeted by the prediction and which the one available experiment tested for and did not observe, requires a gap wide enough to drive both half reactions, band edges positioned to straddle the two potentials, carriers mobile enough to reach the surface before recombining, and a surface that binds intermediates without poisoning. Monolayer fullerene networks satisfy the first three conditions simultaneously in first-principles screening, which is uncommon and is the basis of the original proposal [61]. The fourth condition is where the molecular character of the material becomes an advantage: the exposed cage surface retains the electron-accepting behaviour of C_60_, so photogenerated electrons are drawn onto the network and held there, suppressing the back-transfer that limits many carbon photocatalysts.

Theoretical studies help separate the roles played by the network and the active site. Suo et al. predicted that transition-metal atoms anchored on qHP C_60_ can promote spin-triplet O_2_ activation and CO oxidation through combined charge transfer and spin accommodation [71]. Similarly, Wang et al. identified Cr@graphullerene as a high-loading single-atom ORR candidate in DFT/microkinetic modelling, with a calculated overpotential of 0.389 V and metal loading of 12.6% [89]. Covalent surface functionalisation provides a different route: aryl-diazonium chemistry was experimentally shown to change the band structure and carrier lifetime for visible-light nitrogen reduction [28], and cobaloxime units immobilised on 2D fullerene nanosheets were reported to enhance photocatalytic CO_2_-to-CO conversion [90]. Across these studies, the most transferable design principle is not a single catalytic number but reversible charge accommodation by the cage network together with chemically defined anchoring sites; the reaction-specific activity still belongs to the complete network/active-site assembly.

### 11.2. Photodetection and Optoelectronics

Photodetection is one of the clearest demonstrations that the electronic properties survive solution processing and device fabrication. Electrochemically exfoliated monolayers were integrated into thin-film photodetectors with a reported response from 405 to 1200 nm and peak photocurrent near 850 nm [39]. Rapidly exfoliated mono/bilayers from the 2026 alkali-intercalation/quench route showed about four-fold stronger photocurrent than bulk Mg_4_C_60_ [40]. Multilayer theory further predicts that stacking can enhance visible exciton absorption while increasing transport anisotropy [62].

These device metrics need to be read in context. The reported response spans 405 to 1200 nm with a peak photocurrent near 850 nm and a stable temporal response [39], which places the useful range from the visible into the near-infrared and beyond the nominal optical gap of the material. Sub-gap response of this kind is, in general, characteristic of defect- and interface-mediated absorption; the authors themselves speculate instead that it arises from the delocalised π-conjugated system of the cage network [39]. The mechanism is therefore not established either way, which is advantageous for broadband detection and at the same time means the operating mechanism may not be purely intrinsic. That is a realistic starting point, not a weakness: most successful photodetector technologies exploit such mechanisms, but it does mean the performance should not be attributed solely to the monolayer band structure.

What makes this result more significant than a single demonstration is the material it was achieved on. The detectors were fabricated from electrochemically exfoliated sheets rather than from an individual mechanically exfoliated flake, so the device performance reflects material produced by a route with realistic throughput. The next obstacles include contact resistance, wafer-scale uniformity, ambient stability and reproducibility, together with fullerene-network-specific control of phase and bridge identity, residual Mg and defect chemistry. Establishing whether the polarisation sensitivity predicted from optical anisotropy survives into a fabricated device would test the structure–property chain end to end.

### 11.3. Membranes, Molecular Separation and Storage

The intrinsic corrugation and sub-nanometre free volume of covalent C_60_ sheets create transport channels that are not intrinsic to pristine, non-porous graphene. Chen et al. fabricated ultrathin porous fullerene membranes for organic-solvent separation [91]. In 2026, fullerene–graphene heterochannel membranes combined corrugated C_60_ walls with flat graphene and achieved very high ambient-temperature solvent-vapour flux while maintaining dye rejection [92]. The membrane literature therefore provides an experimentally realised application in which cage geometry, rather than electronic structure alone, is a functional asset.

The membrane results are among the strongest experimental evidence in the field because they are performance measurements on macroscopic assemblies. Stacked porous fullerene nanosheets were measured to deliver organic-solvent fluxes up to two orders of magnitude above comparable membranes at similar rejection [91]. The intended pathways are sub-nanometre lateral channels and in-plane pores; the observed selectivity indicates that these routes dominate, but non-selective defects cannot be assumed absent. The fabricated heterochannel architecture combining fullerene sheets with graphene separates transport functions: graphene supplies low-friction lateral pathways while the fullerene layer supplies molecular-scale selectivity [92]. Electronic-grade perfection is unnecessary, yet large defects and poorly controlled grain boundaries still require suppression because they can bypass the selective channels.

Two structural features distinguish the experimentally demonstrated organic-solvent membranes from graphene-oxide laminates. Their in-plane porosity is intrinsic to the cage packing rather than created only by random defects, and the sheets are covalently bonded internally, limiting the swelling and structural rearrangement that can affect hydrogen-bonded laminates [91,92]. The same pore geometry has motivated two important theoretical extensions. Tong et al. used concentration-gradient molecular dynamics to predict high-permeance H_2_ separation through a quasi-square monolayer fullerene membrane, with strong H_2_/CO_2_ and H_2_/O_2_ selectivity arising from pore-size matching and an entropic funnel effect [93]. Ren et al. combined first-principles calculations and molecular dynamics to predict hydrogen storage in monolayer C_60_ networks through both chemisorption and physisorption, including model-derived internal H_2_ pressures above 116 GPa [94]. Both are class-D application predictions and should remain separate from the experimentally demonstrated solvent-separation results.

Energy storage has also moved beyond prediction. Mei et al. reported qHP-C_60_ as an anode for sodium-ion storage, with a maximum reversible capacity of 279 mAh g^−1^ at 25 mA g^−1^, and as a catalyst in Na–Cl_2_ batteries operating at current densities up to 15,000 mA g^−1^ with 5 wt% catalyst [5]. These are device-level properties of an electrode or catalytic assembly and are therefore class B, but they extend the experimentally demonstrated function of the covalent network beyond the Mg_4_C_60_ lithium-ion boundary case [95].

### 11.4. Sensing, Metal Anchoring, Functionalisation and Biomedicine

Surface chemistry supplies the control needed for these applications. Even in a covalently linked network a substantial fraction of each cage surface remains exposed and available as an adsorption site, with binding governed by dispersion, electrostatics and, in some cases, partial charge transfer. First-principles work predicts qHP/qTP-dependent selectivity toward volatile organic compounds and further tuning by boron doping [96]. BN-doped qTP C_60_ has also been predicted to adsorb H_2_O much more strongly than CO_2_ or N_2_, with adsorption-induced band-gap shifts; this makes humidity an important variable, not a negligible background in any proposed gas-sensing experiment [97]. Intrinsic pore or hole sites between cages provide additional positions for metal clusters: Xu et al. predicted stable Cu4 anchoring in qHP holes, with the calculated band gap reduced to about 0.11 eV while mechanical anisotropy was retained [30], and later experiments showed that monolayer C_60_ networks can support ultrasmall Ir nanoparticles [29].

The molecular chemistry behind these effects is worth stating once, because it explains why the predictions are credible. Fullerene surface chemistry is highly developed: addition reactions at the reactive bonds, and fluorination in particular, alter binding strength and selectivity substantially, through a combination of dispersion, electrostatic and partial-charge-transfer contributions. The same chemistry underlies the covalently linked donor–acceptor assemblies in which a chromophore is tethered to a fullerene and photoinduced electron transfer proceeds to the cage, with the efficiency of charge separation and the rate of recombination controlled by the linker geometry. In a network the individual cage does not lose that function so much as share it: the connectivity provides additional pathways over which a separated charge can delocalise. The caveat is the one raised in Section 9—the bridges consume the most reactive cage positions, so the transferable claim is the mechanism, not the reported molecular yields.

Taken across the application classes, the evidence divides sharply by maturity. Photodetection on electrochemically exfoliated material and organic-solvent separation through porous fullerene membranes are device-level demonstrations on real material [39,91]. Pure-water photocatalysis and several metal-supported electrocatalytic reactions are experimental laboratory-scale demonstrations [29,84,85,86,87,88], while hydrogen separation, hydrogen storage, VOC sensing, single-atom ORR and several anchoring concepts remain theoretical [89,93,94,96]. An additional boundary case is Mg_4_C_60_: the metal-containing covalently bridged layered precursor has been demonstrated as a stable Li-ion anode framework in which the bridges suppress fulleride dissolution [95]. It is relevant to the chemistry and robustness of linked C_60_ frameworks, but it is not the pristine demetallated 2D carbon network and is therefore retained as a derivative benchmark rather than merged into the core property set.

Biomedical use is the newest and narrowest application direction, but it is no longer purely hypothetical. Lv et al. experimentally compared HP-2D-C_60_ and TP-2D-C_60_ nanosheets and reported strong NIR-II photothermal activity for the tetragonal material, including in vitro and in vivo antitumour tests and a photothermal conversion efficiency of 43.02% [31]. That result is direct evidence for photothermal functionality of a covalent C_60_ network. It should not, however, be extrapolated to the wider molecular-fullerene biomedical literature: long-term biocompatibility, clearance, structural persistence in biological media and the extent to which the photochemistry of isolated C_60_ survives polymerisation remain open questions. Table 7 collects the application-directed studies together with the material status behind each result.

## 12. Cross-Property Benchmark: What Is Actually Established?

A critical synthesis of the literature produces a more nuanced picture than the common ‘strong semiconductor with low thermal conductivity’ slogan. The qHP network is mechanically much stiffer than van der Waals fullerite, but its intrinsic in-plane stiffness remains below graphene and is strongly anisotropic. Its strength and fracture strain are more temperature-sensitive than its small-strain stiffness. Electronic semiconducting behaviour is robust across experiment and theory, but the numerical gap varies by observable and topology. Strong excitonic binding and polarisation anisotropy are well supported theoretically, while quantitative exciton energies remain sensitive to screening conventions. Thermal conductivity is the least settled major property, with a nearly two-order-of-magnitude spread among credible methodologies. Tribology is promising but currently predominantly computational. Applications, in contrast, have moved rapidly into direct experiments, especially catalysis, photodetection and membranes.

Grouping the evidence by maturity rather than by property clarifies where the field stands. Two points have strong direct experimental support and independent theoretical context: a covalent qHP monolayer can be isolated, and it is a semiconductor with an anisotropic transport gap near 1.6 eV. A third conclusion—that covalent linking yields stiffness far above molecular fullerite, with normalised theoretical qHP values of order 10^2^ N m^−1^—is supported by convergence among calculations on experimentally anchored structures and by bulk/few-layer contrasts, but still lacks direct monolayer mechanical measurement. Strong mechanical anisotropy, strongly bound excitons and pronounced temperature sensitivity of strength are supported by multiple calculations but also lack direct monolayer validation. The rapidly growing application literature adds a separate form of experimental maturity: the network functions as an electronically active support, membrane, energy-storage material and chemically modifiable platform, although the reported activities belong to complete assemblies rather than pristine C_60_ alone.

Three remain genuinely unsettled. The thermal conductivity is the clearest case, with published values for nominally the same monolayer differing by more than an order of magnitude depending on the interatomic model and the treatment of anharmonicity, and no direct measurement on an isolated covalent monolayer to arbitrate between them. The transport regime is the second: whether carriers in a real sheet are band-like or polaronic changes the predicted mobility by a large factor and is not resolved. The third is the behaviour of real, flawed, supported material, since essentially every quantitative result in the literature refers to a defect-free freestanding sheet while every experimental sample is finite, supported and imperfect.

Reading the field this way also explains why the apparent contradictions cluster where they do. Quantities that do not require an arbitrary dimensional normalisation, such as lattice parameters and gap energies, are generally easier to compare, although they remain structure- and method-dependent. The largest quantitative disagreements occur when an additional convention or model enters the reported value itself: effective thickness for GPa stiffness, interatomic potential and transport algorithm for thermal conductivity, transport regime for mobility, or dielectric/slab treatment for exciton binding. The disagreement is therefore not evidence of poor work; much of it reflects a reporting culture that has not yet converged on a common set of descriptors and conventions.

The open questions can be translated into decisive experiments. For stiffness, nanoindentation on a suspended covalent monolayer would yield the two-dimensional modulus directly in N m^−1^, with no thickness convention, and could provide intrinsic strength if taken to failure. For thermal transport, a suspended-membrane or optothermal Raman measurement on an isolated monolayer would arbitrate between predictions differing by more than an order of magnitude. For charge transport, temperature-dependent four-terminal field-effect mobility, combined with contact-resistance correction, gate-bias dependence and comparison with competing transport models, would help distinguish predominantly band-like from activated or polaronic behaviour. These measurements are established for other 2D materials and have become feasible as flakes of tens of square micrometres are produced [39,40]. The principal quantitative disagreements are now experimentally limited. Table 8 sets out the benchmark property by property, while Figure 5 maps how bridge chemistry fixes topology and directional response.

## 13. Recommended Reporting Standard for Future C_60_-Network Studies

The field is now large enough that reproducibility requires a minimum reporting set. The following items would remove most of the ambiguity identified in this review.

For mechanical work the minimum set is short. Report the two-dimensional elastic constants and Young’s moduli in N m^−1^ as the primary quantities, since these are what a calculation determines. If a stress in GPa is also quoted, state the thickness used to obtain it in the same sentence, so that any reader can invert the conversion. State the crystallographic axes explicitly with reference to the bridge orientation, not to an unlabelled x and y, which would have prevented the sign conflict in reported anisotropy discussed in Section 6.3. Declare which stability criterion a claim of stability refers to. And when a potential is used, report at least one property computed with a second potential on the identical geometry, so that model sensitivity is measured rather than asserted.

For transport and optical work the analogous requirements are to state the transport model assumed when a mobility is quoted, to report the slab geometry and Coulomb treatment alongside any exciton binding energy, and to give the layer count and stacking registry alongside any thermal conductivity, since the monolayer and bilayer values differ systematically [79]. None of this constrains what may be calculated or measured; it constrains only what must accompany the number. Adopted consistently, it would allow the next generation of studies in this field to be compared directly, which the present generation largely cannot be.

A final recommendation concerns the reporting of structure itself. Because properties in this family are controlled by bridge motif, not by cage packing, a structural specification that gives only the lattice parameters and the space group is insufficient to reproduce a calculation. The minimum useful description states which bridge motif occupies which lattice direction, gives the number of covalent links per cage, and supplies the relaxed atomic coordinates. Several of the inconsistencies traced in this review would have been impossible had that information accompanied every reported structure, since it would immediately reveal whether two studies claiming to model the same phase were in fact modelling the same connectivity.

- Structure: provide atomic coordinates or a persistent structure file, lattice vectors, number of cages per cell, exact inter-cage bond types and molecular orientation; do not rely on “qHP” or “qTP” alone.
- Mechanics: report the 2D elastic tensor in N m−1, loading direction tied to lattice vectors, strain definition, strain rate, temperature, boundary conditions, sample size and potential/DFT functional. If GPa is used, state t explicitly. Where an empirical potential is used, state the element assignment taken from its parameter file as well as the potential itself: multi-element reactive parameter sets contain more than one carbon type, intended for different bonding environments, and which one is selected is almost never reported.
- Fracture: distinguish first-bond-breaking, peak stress, loss of connectivity and complete rupture; report whether cages themselves open or only inter-cage bridges fail.
- Electronic structure: identify the gap type and functional/many-body approximation. Report whether the gap is direct/indirect and specify k-points for VBM/CBM where appropriate.
- Optics: report quasiparticle and optical gaps separately, the physical dielectric environment, dielectric treatment, k-point and vacuum convergence, Coulomb truncation/screening approach and polarisation direction.
- Thermal transport: state layer count, direction, effective thickness if a volumetric κ is reported, potential, transport algorithm, cell-size convergence, simulation time and defect density.
- Multilayers/interfaces: state stacking registry, interlayer distance, whether interlayer bonding is covalent or van der Waals, and whether the reported quantity is an intrinsic layer property or an effective composite response.
- Evidence language: explicitly label predictions as theoretical and do not use “demonstrated” for class-D structures or application concepts without experimental validation.

## 14. Conclusions and Outlook

Two-dimensional C_60_ networks are cluster-based carbon crystals rather than graphene assembled from buckyballs, and their mechanical, electronic, optical and thermal anisotropies share one structural origin: the cage modes, the curvature, the inequivalent junctions and the stacking degrees of freedom of the covalent bridges that join the cages. Once the thickness convention is removed, the apparent 4.4-fold spread in reported quasi-hexagonal stiffness, 49.6–218.5 GPa, narrows to roughly 2.0-fold in thickness-independent terms, 93–189 N m^−1^—because the two study populations differ, this is a benchmarking diagnostic rather than a meta-analytic effect size. The robust result is an in-plane stiffness of order 10^2^ N m^−1^ with strong bond-orientation anisotropy, while strength and fracture remain markedly more sensitive to temperature, defects and the interatomic description.

The quasi-hexagonal network, the one phase whose structure is experimentally established, behaves as a semiconductor in the devices built from it [39] and is computed to be strongly anisotropic. The transport, optical and quasiparticle gaps are different physical quantities, while the Kohn–Sham and hybrid eigenvalue gaps are properties of a calculation rather than observables at all, so none of the five may be compared with another as though they were one number.

Uncertainty is not evenly distributed across the properties reviewed here. Thermal transport is the least mature: credible monolayer predictions still span from a few to more than 100 W m^−1^ K^−1^ [52,53,82], the only experimentally anchored value refers to micrometre-thick layered material, and method, topology, direction and layer count must therefore accompany any quoted conductivity. Beyond that, inconsistent reporting continues to generate disagreement that is not physics: unstated thickness conventions make values in GPa unrecoverable, non-standard axis labels can reverse an apparent anisotropy, and the word ‘stable’ is used for four criteria that answer four different questions. Most models still describe ideal sheets, so computed strengths are upper bounds rather than predictions for real material.

The experimental bottleneck has correspondingly shifted from proving existence to achieving control. Scalable exfoliation, rapid intercalation-assisted processing and continuous-flow sheet formation [39,40] now make the decisive measurements feasible, and three of them would settle more than another round of prediction: thickness-independent monolayer mechanics, a direct monolayer thermal conductivity, and temperature-dependent four-terminal mobility with contact-resistance correction. On the computational side the most valuable exercise is a controlled cross-method thermal benchmark on identical quasi-hexagonal coordinates, temperature and transport protocol.

The significance of C60 networks is unlikely to lie in outperforming graphene on a single figure of merit. Their stronger contribution is to show that a molecular cage can serve as the repeating unit of a periodic covalent solid, combining tunable semiconducting response, directional mechanics and optics, intrinsic porosity, architecture-dependent thermal transport, energy storage and chemically triggered, coordination-mediated disassembly. The next advance should come from structurally identified materials, direct measurements, and comparable calculations, supported by the reporting standard proposed in Section 13.

## Figures and Tables

**Figure 1:**
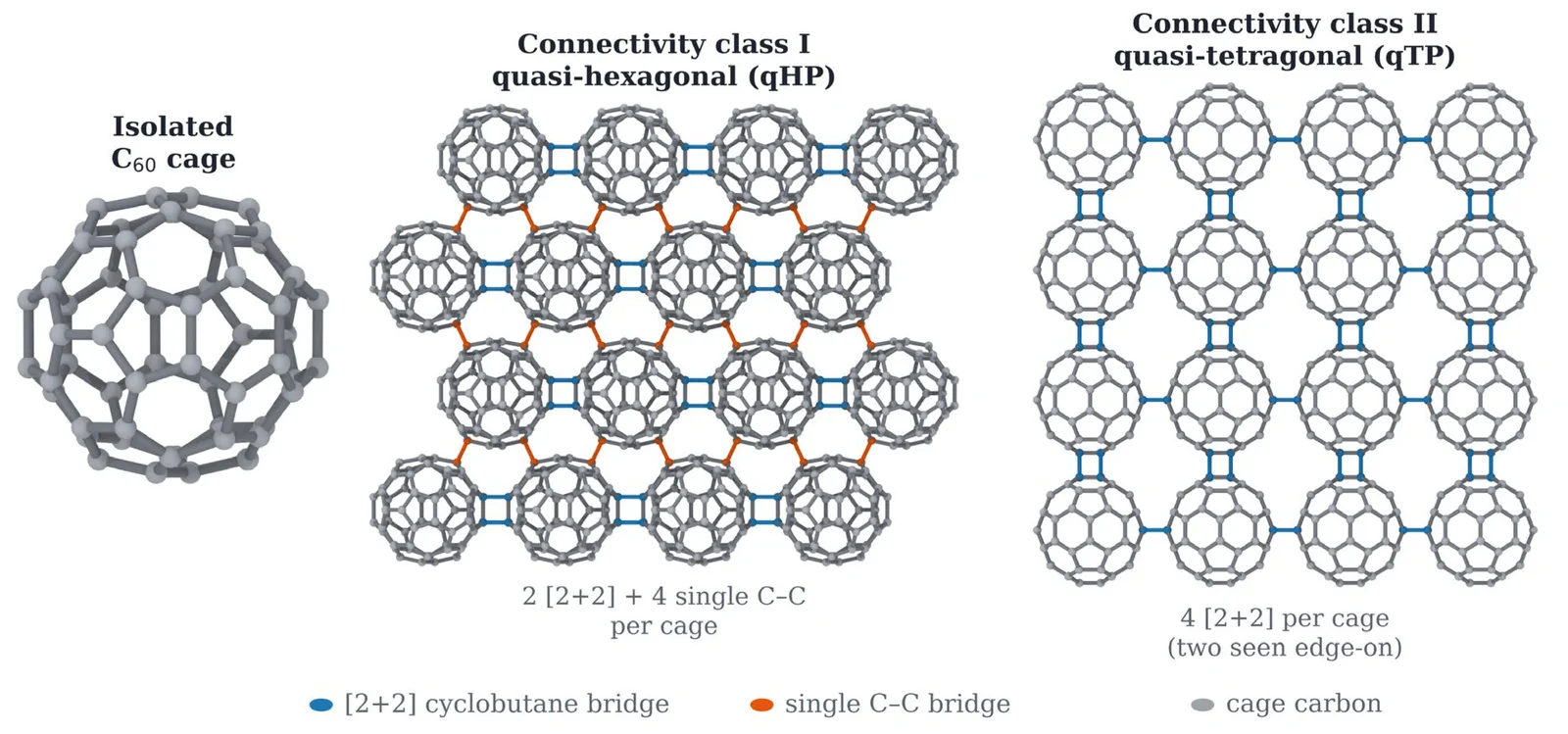
Comparison of an isolated C_60_ cage and two representative two-dimensional connectivity classes associated with quasi-hexagonal (qHP) and quasi-tetragonal (qTP) C_60_ networks. All three panels are rendered from the qHP and qTP monolayer structures used in this work, with [2+2] cyclobutane bridges in blue, single C–C bridges in orange and cage carbon in grey. Bridge colour is assigned from inter-cage connectivity rather than from the projection: viewed along the surface normal, two of the four qTP bridges present their cyclobutane ring face-on and two are seen edge-on, so the number of squares visible in the image is not the number of [2+2] bridges.

**Figure 2:**
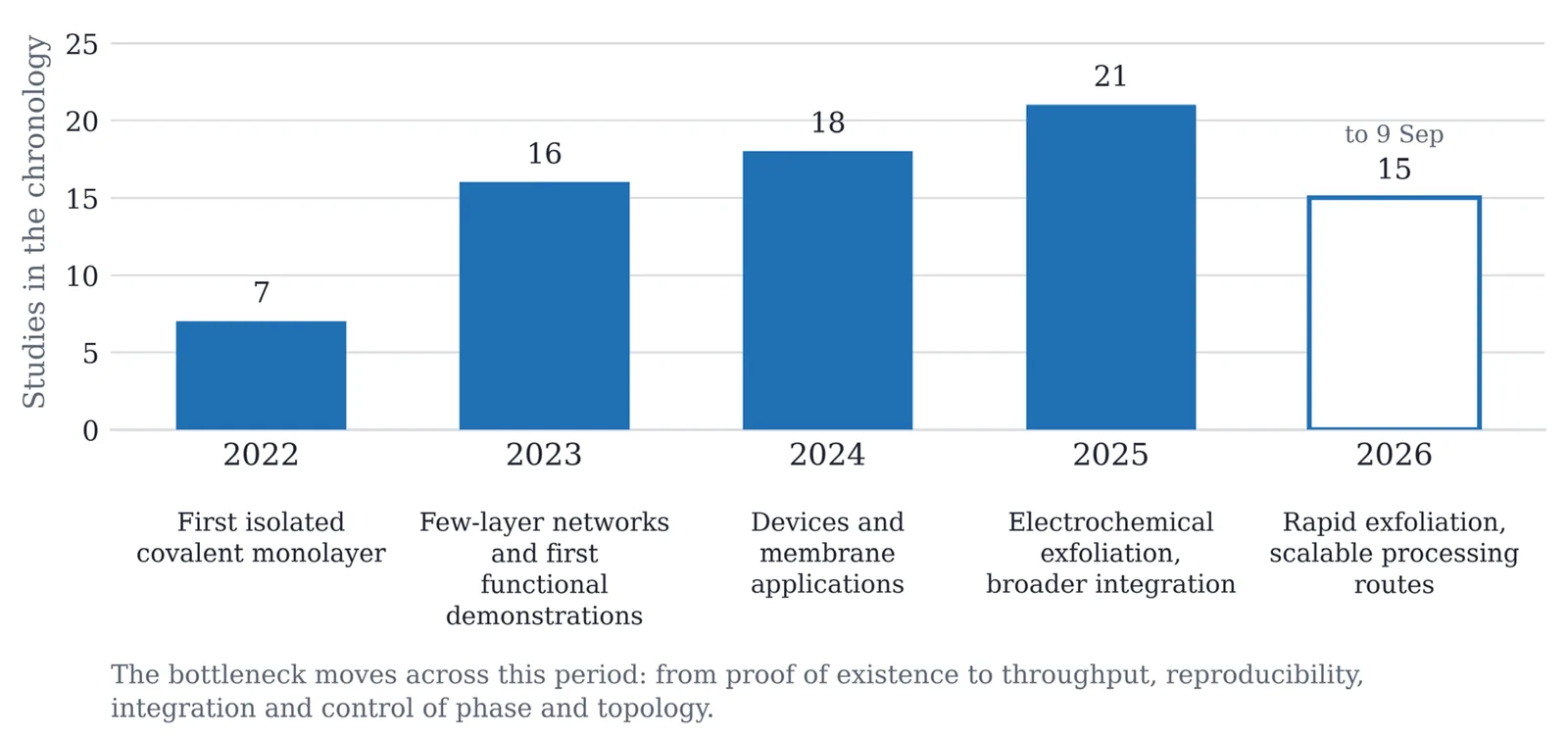
Experimental progression from the first isolated monolayer to scalable exfoliation and emerging 2026 processing routes. Bars count the studies listed under each year in the working corpus of Appendix A (Table A1); the 2026 bar is drawn open because that year is partial, the verified scope closing on 9 September 2026. The timeline emphasises a change in bottleneck: from proof of existence to throughput, reproducibility, integration and control of phase/topology.

**Figure 3:**
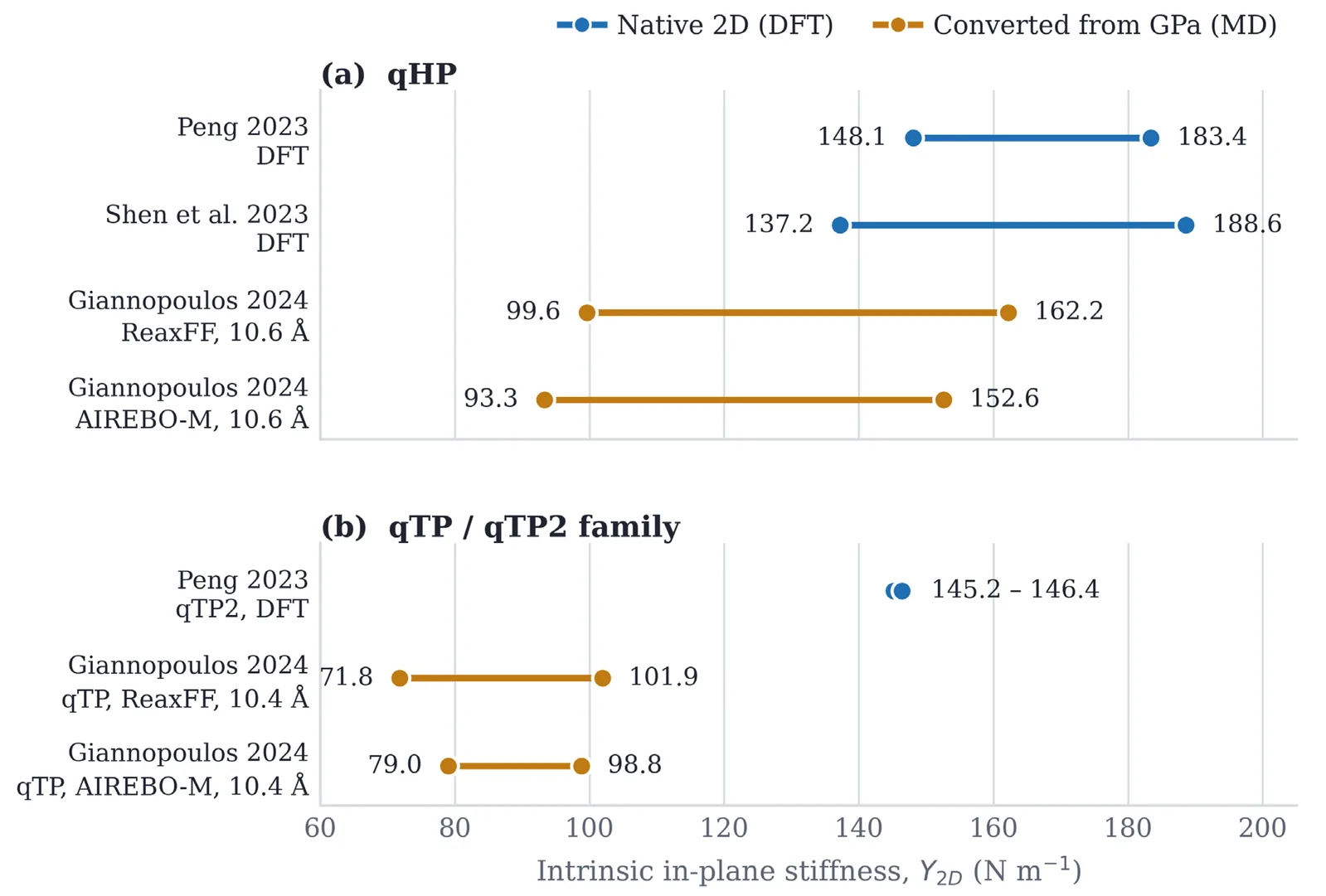
Thickness-independent comparison of representative recoverable qHP and qTP/qTP2 in-plane stiffness values. The panels separate (**a**) the qHP phase from (**b**) the qTP/qTP2 family; within each panel, colour separates native two-dimensional results from values converted using the effective thickness declared in the corresponding study. Each horizontal segment joins the two reported in-plane directional endpoints without imposing a common crystallographic axis convention across studies. The qTP/qTP2 entries are presented as a family comparison rather than as an identical topology; the mechanically unstable qTP1 result is excluded from quantitative benchmarking. GPa-only studies without a recoverable thickness are excluded rather than normalised using an assumed value [13,19,51].

**Figure 4:**
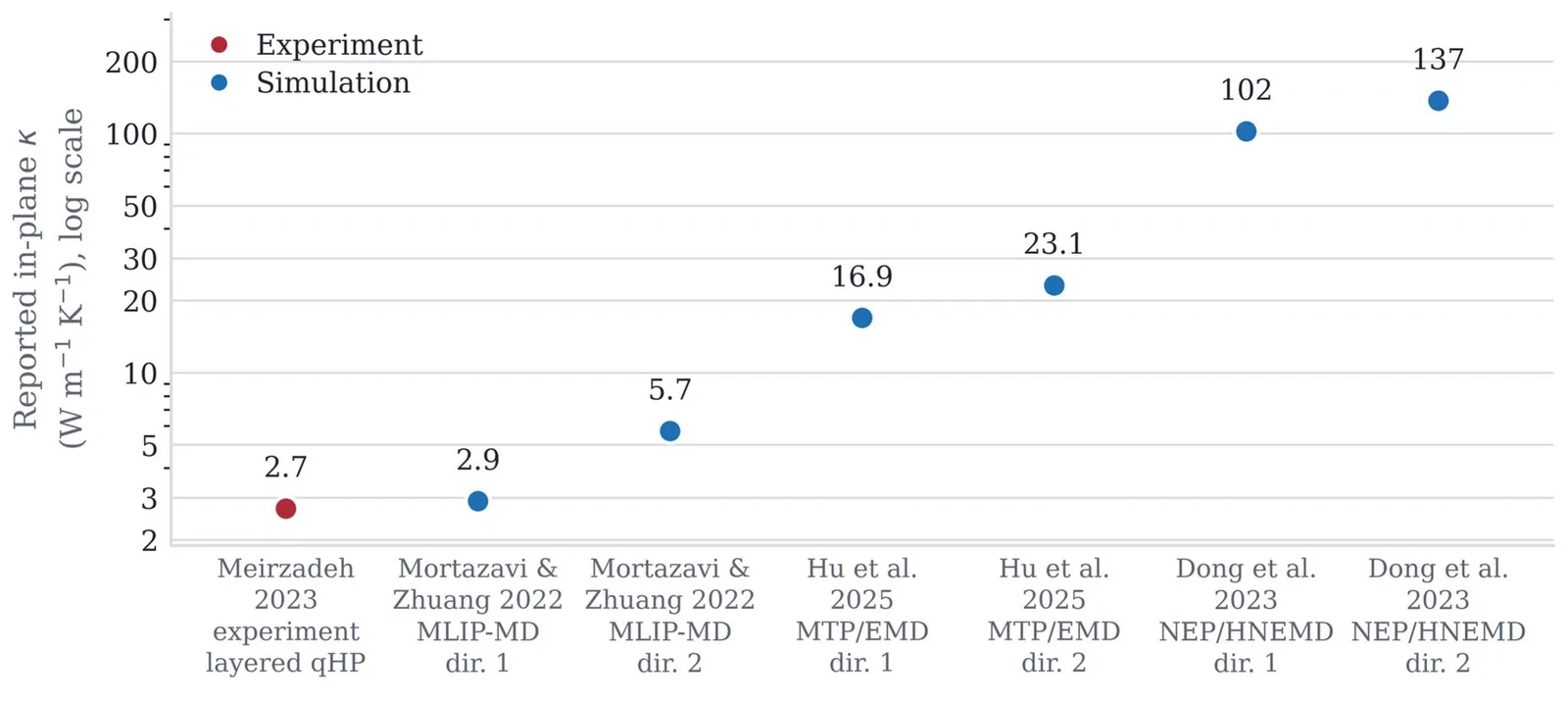
Representative published thermal-conductivity values illustrating the unresolved method dependence. The logarithmic plot spans the experimentally anchored micrometre-thick layered baseline and monolayer simulations from low-conductivity machine-learning interatomic potential (MLIP) results to the highest neuroevolution potential (NEP) prediction. Direction 1 and direction 2 denote the two in-plane directions as each study reported them; no common crystallographic axis convention is assumed across studies. The values should not be collapsed into a single characteristic conductivity; method, interatomic model, transport algorithm, direction and layer count must accompany any quantitative comparison [4,52,53,82].

**Figure 5:**
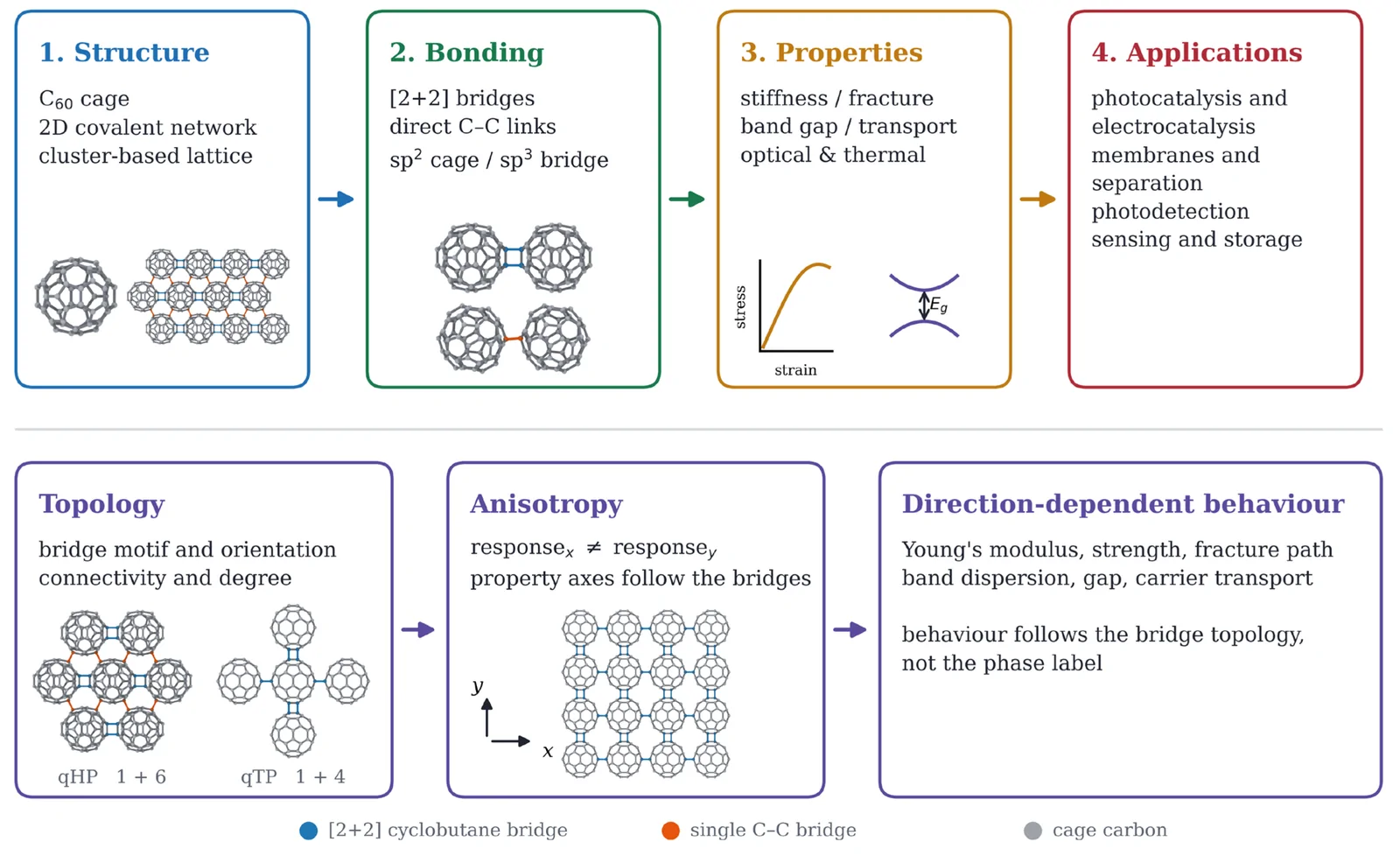
Conceptual structure–bonding–property–application map of two-dimensional C60 fullerene networks. The upper pathway illustrates how the cluster-based C60 architecture and inter-cage bonding motifs influence mechanical, electronic, optical and thermal properties and, ultimately, application-relevant functionality. The lower pathway highlights the mechanistic relationship between network topology, lattice anisotropy and direction-dependent mechanical and electronic behaviour. Every cage and network panel is rendered from the qHP and qTP monolayer structures themselves: [2+2] cyclobutane bridges in blue, single C–C bridges in orange, cage carbon in grey. The qHP junction shows one cage with its six neighbours and the qTP junction one cage with its four. The stress–strain and band-structure insets remain schematic and stand for classes of response, not for any measured dataset.

**Table 1:** Evidence classes used throughout the review, ordered by evidential proximity to the target C60 network. Quantitative conclusions are weighted toward classes A–C; class D structures are valuable for design principles but must not be pooled numerically with experimentally realised qHP material. A study reporting results of more than one kind carries a slash label (A/B, C/D); classes are assigned at result level, not per paper.

Class	Evidential Proximity to the Target C60 Network	Use
A	Direct experimental evidence on pristine covalent C60 networks, including synthesis and intrinsic or near-intrinsic characterisation	Quantitative
B	Experimental devices, supported materials, functionalised sheets and other derivatives whose measured response includes an interface	Quantitative
C	Theory or simulation on an experimentally anchored C60-network topology, whatever the method	Quantitative
D	Theory on an unrealised network topology, chemical derivative or functionality, with no corresponding material established	Design logic only; never pooled numerically with A–C
E	Molecular and bulk benchmarks, reviews and perspectives, used for context and not as direct property evidence	Context only

**Table 2:** Experimental progression of C_60_-network synthesis and processing, from the first isolated monolayer to scalable exfoliation and emerging 2026 routes. Evidence classes follow the definitions of Section 2.2.

Study	Material/Route	What Was Established	Evidence
Hou et al. (2022) [3]	Mg-templated precursor > monolayer qHP; few-layer qTP	Experimental qHP monolayer with anisotropic semiconducting transport; few-layer qTP also reported	A
Meirzadeh et al. (2023) [4]	few-layer covalent network	Covalent fullerene network persists in few-layer form; thermal baseline	A
Zhang et al. (2025) [39]	electrochemical exfoliation	~52.5 μm^2^ monolayer flakes; ~83% yield; gram-scale (A); solution-processed photodetectors (B)	A/B
Yuan et al. (2026) [40]	alkali intercalation + quench	mono/bilayers released in <10 min (A); ~4× photocurrent versus bulk precursor (B)	A/B
Alotaibi et al. (2026) [41]	high-shear continuous flow	covalent rectangular C_60_ sheets ~10–15 nm thick	A
Lv et al. (2026) [31]	HP-2D-C_60_ and TP-2D-C_60_ nanosheets	hexagonal- and tetragonal-phase synthesis (A); TP NIR-II photothermal response (B)	A/B
Jia et al. (2026) [6]	W/Ni coordination of 2D fullerene frameworks	inter-cage σ-bond scission and release of intact C60; Mg intercalation promotes junction opening, and removing Mg^2+^ suppresses it	B/C

**Table 3:** Thickness-independent restatement of representative qHP and qTP/qTP2 in-plane stiffness values. Native two-dimensional results and values converted with the thickness declared by the corresponding study are listed together; GPa-only studies without a recoverable thickness are marked as not recoverable rather than back-calculated from an assumed value.

System/Source	As Published	Thickness Used	Normalised Y_2D_	Interpretation
qHP, Peng [13]	148.1/183.4 N m^−1^	native 2D	148.1/183.4 N m^−1^	directly comparable
qHP, Shen [19]	tensor in N m^−1^	native 2D	~137.2/188.6 N m^−1^	derived from C_ij_
qHP, Giannopoulos et al. ReaxFF [51]	~153/94 GPa	10.6 Å	~162/100 N m^−1^	recoverable
qHP, Giannopoulos et al. AIREBO-M [51]	~144/88 GPa	10.6 Å	~153/93 N m^−1^	recoverable
qTP2, Peng [13]	146.4/145.2 N m^−1^	native 2D	146.4/145.2 N m^−1^	nearly isotropic; structurally specific qTP2
qTP, Giannopoulos et al. ReaxFF [51]	~98/69 GPa	10.4 Å	~102/72 N m^−1^	recoverable; qTP model
qTP, Giannopoulos et al. AIREBO-M [51]	~95/76 GPa	10.4 Å	~99/79 N m^−1^	recoverable; qTP model
qHP, Tromer [16]	~49.6/61.1 GPa	not stated	not recoverable	do not normalise by assumption
qHP, Ribeiro Junior [43]	175.9 (x)/218.5 (y) GPa	not recoverable	not recoverable	retain as published; note BOTH directions
qHP, Yu et al. [12]	191.6 GPa	not stated	not recoverable	freestanding MD baseline
qHP + graphene encapsulation [12]	531.4 GPa	composite	not comparable	effective composite modulus

**Table 4:** Compendium of reported mechanical properties of isolated cages, fullerite, covalent C_60_ networks and supported composites, in the units used by each source. The table is a record, not a ranking, and should be read against the normalisation of Section 6.3 and the evidence classes of Section 2.2.

System/Material	Method	Young’s Modulus	Strength/Poisson	Key Finding	Source
Isolated C_60_ cage	DFT-MD compression	—	elastic to ε = 0.48 ± 0.02	Three regimes: elastic → 30–32 nN plateau with stochastic bond rupture → irreversible beyond ε = 0.75 ± 0.02	Pizzagalli 2022 [48]
Fullerite film, fcc (110) texture	Nanoindentation	14.1 GPa	H = 0.42 GPa	Cohesion dominated by van der Waals interactions with a small covalent admixture; matches the 14.7 GPa calculated for the (110) direction	Rudchenko et al. 2013 [46]
Monomeric C_60_ film	Nanoindentation	7.6 ± 3.6 GPa	H = 0.3 ± 0.1 GPa	Weak inter-cage interaction typical of monomeric fcc C_60_	Dorner-Reisel et al. 2023 [47]
qHP monolayer	DFT, first baseline	49.6/61.1 GPa	anisotropic	First dedicated DFT study; thickness not stated, so the value cannot be normalised	Tromer et al. 2022 [16]
qHP monolayer	DFT, full elastic tensor	C_11_ = 140.2, C_22_ = 192.6 N m^−1^	C_12_ = 23.9, C_66_ = 63.5 N m^−1^	Born–Huang criteria satisfied; stiffness rises monotonically from 0° to 90°	Shen et al. 2023 [19]
qHP monolayer	DFT, phase comparison	148.1/183.4 N m^−1^	ν = 0.120/0.149	Stiffest dense phase; the qTP2 entry below is the more balanced pair	Peng 2023 [13]
qTP2 monolayer	DFT	146.4/145.2 N m^−1^	ν = 0.154/0.153	Nearly isotropic; balanced load paths, unlike qTP1	Peng 2023 [13]
qTP1 monolayer	DFT	5.4/123.5 N m^−1^	ν = −0.010/−0.225	C_66_ = −0.2 N m^−1^ fails the Born criteria: the auxeticity is an instability signature, not an exploitable property	Peng 2023 [13]
Five covalent network patterns	DFT screening	131.7–208.0 N m^−1^	—	Spread set by bridging-bond density and orientation, not by cage chemistry	Yan et al. 2025 [14]
qHP monolayer	ReaxFF MD	175.9 (x)/218.5 (y) GPa	—	Linear crack propagation; both directions must be quoted, and this study puts the stiff axis along y	Ribeiro Junior et al. 2022 [43]
qTP monolayer	ReaxFF MD	100.7 (x)/133.5 (y) GPa	—	Fracture mode reported inconsistently within the source; topology-dependent crack path, while damage tolerance remains unquantified	Ribeiro Junior et al. 2022 [43]
qHP monolayer	ReaxFF MD, targeted parameterisation	~153/94 GPa (t = 10.6 Å)	—	Normalises to ~162/100 N m^−1^	Giannopoulos et al. 2024 [51]
qHP monolayer	AIREBO-M MD	~144/88 GPa (t = 10.6 Å)	—	Normalises to ~153/93 N m^−1^—about 6% from the ReaxFF result under a fixed protocol	Giannopoulos et al. 2024 [51]
qTP monolayer	ReaxFF/AIREBO-M MD	~98/69 and ~95/76 GPa (t = 10.4 Å)	—	Lower than qHP under every methodologically consistent comparison	Giannopoulos et al. 2024 [51]
qHP/qTP nanosheets	ReaxFF MD, thermomechanical fracture	direction- and temperature-dependent	—	Fracture behaviour and crack initiation of fullerene nanosheets under combined thermal and mechanical loading	Giannopoulos 2024 [54]
qHP monolayer at 1 K	MLIP-MD	—	7.0/8.8 GPa; εf ≈ 0.07	Ground-state upper bound on strength	Mortazavi & Zhuang 2022 [52]
qHP monolayer at 300 K	MLIP-MD	—	3.3/4.2 GPa; εf ≈ 0.029	~53% strength loss against 1 K; microscopic contribution of cage/junction modes remains to be isolated	Mortazavi & Zhuang 2022 [52]
qHP monolayer	DFT + neuroevolution MD	direction-dependent	—	Fragility traced to inhomogeneous deformation between rigid cages and soft bridges	Ying et al. 2023 [18]
qHP monolayer	Classical MD: T, size, defects	reduced by defects	reduced by defects	Stiffness, strength and toughness all fall with defect density and temperature	Ajori et al. 2024 [50]
qHP monolayer	DFT + MD anisotropy study	direction-dependent	strongly anisotropic	The full polar plot is required; the modulus extrema lie on the crystallographic axes while the strength extrema do not	Zhao et al. 2023 [21]
Porous C_60_ lattices	DFT + MLIP	up to ~300 GPa (t = 8.8 Å)	up to ~50 GPa	Normalises to ~264 N m^−1^ and exceeds every dense phase: bond orientation beats atomic density	Mortazavi 2023 [15]
C_36_-based network	DFT + MLIP	266 GPa (t = 6.06 Å)	auxetic	Normalises to ~161 N m^−1^; the published GPa value does not imply larger intrinsic 2D stiffness than qHP	Mortazavi et al. 2023 [11]
qHP freestanding	MD	191.6 GPa	—	Freestanding baseline for the encapsulation comparison	Yu et al. 2023 [12]
qHP + graphene encapsulation	MD	531.4 GPa	—	×2.8; a composite structural response, not an intrinsic monolayer property	Yu et al. 2023 [12]
qTP freestanding → encapsulated	MD	134.7 → 504.1 GPa	—	×3.7; defects in the C_60_ layer become almost irrelevant once encapsulated	Yu et al. 2023 [12]

**Table 5:** What each reported electronic quantity can and cannot be used to conclude. Reported electronic and charge-transport values for C_60_ networks are bound to the method and structural model that produced them, so that quantities belonging to different observables are not compared directly; the final column states the admissible conclusion for each row.

Study	Material/Method	Electronic Quantity	Critical Interpretation
Hou et al. [3]	experimental qHP	transport/electronic gap ~1.6 eV	experimental reference, not a universal band gap
Yu et al. [17]	DFT qHP-like	direct gap ~1.55 eV	structure- and functional-specific
Peng [61]	hybrid DFT + optical treatment	phase-dependent ~1.67–1.88 eV	appropriate for photocatalytic band-edge analysis
Yan et al. [14]	DFT, five phases	0.85–1.55 eV	topology controls gap and anisotropy
Capobianco et al. [68]	quantum electron transfer	polaron binding ~0.1 eV	mobility enhancement from stronger inter-cage coupling
Khan et al. [26]	spin-DFT impurities	spin-dependent gaps ~0.43–1.5 eV	predicted half-semiconducting functionality
Cassiano et al. [67]	Holstein–Peierls qHP transport model	large polarons; anisotropy >= 50%; ~0.5–1.5 nm ps^−1^	independent support for anisotropic polaronic transport
Pereira Junior et al. [27]	PBE-DFT endohedral qHP	N: intragap semiconducting states; Ce/Sr: filling-dependent metallicity	endohedral electronic/optical tuning; theoretical
Yadav [24]	DFT qTP2 + BN co-doping	PBE/HSE gap and transport tuning	theoretical dopant-dependent electronic/optical response
Yadav et al. [25]	DFT + classical qHP BN doping	dopant-dependent gap, conductivity and mechanics	qHP-specific theoretical doping benchmark
Li & Sun [60]	DFT bulk + qHP/qTP monolayers	band/charge-density and optical anisotropy	bridge-localised electronic density; strong visible/NIR absorption; qTP polarisation dependence
Chernozatonskii & Kochaev [64]	DFT C_60_-polymer/h-BN bilayer	substrate-modified electronic/optical response	supported heterostructure; do not merge with freestanding gap values
Suo et al. [71]	DFT TM@qHP C_60_	spin-dependent O_2_ activation	predicted spin-sponge/electron-reservoir functionality; no experimental magnetic order
Xu et al. [72]	real-time VG-DFTB qHP C_60_ in solvent	electron–hole recombination dynamics	water strongly affects molecular C_60_ but has comparatively little effect on qHP dynamics
Peng & Pizzochero [66]	DFT qHP/qTP-derived nanoribbons	width/edge-dependent gaps and edge states	1D derivative; useful boundary case, not a 2D bulk property

**Table 6:** Representative reported thermal conductivities near 300 K, with the system and method each value belongs to and the conclusion it supports.

Source	System/Method	Representative κ at ~300 K	What Should Be Concluded
Meirzadeh et al. [4]	experiment, few-micrometre-thick layered qHP C_60_	~2.7 W m^−1^ K^−1^	experimentally anchored layered-material baseline; not an isolated-monolayer value
Mortazavi & Zhuang [52]	qHP, MLIP-MD	~2.9/5.7	low and anisotropic
Dong et al. [53]	qHP, NEP/HNEMD	~102/137	very high prediction; unresolved discrepancy
Hu et al. [82]	qHP, MTP/EMD	~16.9/23.1	intermediate; topology controls κ
Hu et al. [82]	four C_60_ networks, MTP/EMD	~9.2–34.4	network design tunes magnitude/anisotropy
Yang et al. 2026 [80]	overlapping qHP interfaces, atomistic simulation	vacancy-sensitive	thermal resistance depends on overlap and defects
Tea et al. [78]	pristine single-crystal C_60_ fullerite, experiment	~0.4 W m^−1^ K^−1^ at room temperature	molecular-crystal baseline; weak intermolecular thermal coupling
Smontara et al. [83]	hard carbon transformed from compressed C_60_, experiment	~5.5 W m^−1^ K^−1^ at room temperature	transformed-carbon benchmark; not a covalent 2D network

**Table 7:** Application-directed studies of C_60_ networks, showing the material status behind each reported result: experimentally realised, supported or functionalised derivative, or hypothetical polymorph.

Application	Representative Study	Material Status	Key Result/Significance
Pure-water photocatalysis	Wang et al. 2023 [84]	experimental few-layer	H_2_/H_2_O_2_ evolution ~91/116 μmol g^−1^ h^−1^ without sacrificial reagent
CO_2_ electrocatalysis	Zhang et al. 2024 [85]	bi/few-layer C_60_	>90% FE to HCOOH over broad potential window
Organic-solvent membranes	Chen et al. 2024 [91]	experimental porous C_60_ nanosheets	high-flux selective solvent separation
Photodetection	Zhang et al. 2025 [39]	experimental monolayer	405–1200 nm photoresponse; scalable solution processing
HER catalyst support	Huang et al. 2025 [29]	Ir/monolayer graphullerene	η10 = 18 mV; 16.54 mV dec^−1^; long durability
N_2_ photoreduction	Wang et al. 2025 [28]	aryl-functionalised 2D C_60_	covalent chemistry tunes gap and carrier lifetime
NIR-II photothermal therapy	Lv et al. 2026 [31]	experimental covalent phases	tetragonal phase; 43.02% photothermal efficiency
CO_2_ photoreduction	Wang et al. 2026 [90]	C_60_@cobaloxime	CO 18.1 μmol g^−1^ h^−1^; 96.3% selectivity
Heterochannel membranes	Cai et al. 2026 [92]	C_60_–graphene composite	ultrahigh solvent-vapour flux with dye rejection
Humidity/gas sensing	Yadav 2026 [97]	BN-doped qTP C_60_, theoretical	H_2_O adsorption dominates CO_2_/N_2_; band-gap shifts highlight humidity sensitivity
Ammonia oxidation (AOR)	Chen et al. 2025 [86]	Pt/polymeric C_60_ network, experimental	118 A gPt^−1^ mass activity; electron-buffering support
Oxygen evolution (OER)	Chen et al. 2025 [87]	Ir/C_60_NET, experimental	237 mV overpotential; >600 h at 10 mA cm^−2^
Alkaline HER	Wang et al. 2025 [88]	Ru/few-layer covalent C_60_, experimental	24 mV at 10 mA cm^−2^; ~8× intrinsic activity vs. Ru NPs
Oxygen reduction (ORR)	Wang et al. 2025 [89]	Cr@graphullerene, theoretical	0.389 V calculated overpotential; 12.6% metal loading
H_2_ gas separation	Tong et al. 2023 [93]	quasi-square monolayer, theoretical MD	high H_2_ permeance/selectivity predicted for H_2_/CO_2_ and H_2_/O_2_
Hydrogen storage	Ren et al. 2023 [94]	monolayer C_60_ network, theory + MD	combined chemisorption/physisorption; predicted internal H_2_ pressure > 116 GPa
Li-ion storage (boundary)	Wang et al. 2025 [95]	Mg_4_C_60_ layered covalent framework, experimental	covalent bridges suppress fulleride dissolution; derivative/precursor benchmark
CO oxidation	Suo et al. 2024 [71]	TM@qHP C_60_, theoretical	0.3–0.5 eV reaction barriers; predicted spin-sponge effect
Sodium storage and Na–Cl_2_ conversion	Mei et al. 2026 [5]	qHP-C_60_ electrode/catalyst assembly, experimental	279 mAh g^−1^ at 25 mA g^−1^; Na–Cl_2_ operation up to 15,000 mA g^−1^ with 5 wt% catalyst

**Table 8:** Cross-property benchmark: the best-established conclusion, the dominant remaining uncertainty and the evidence maturity of each property class.

Property	Best-Established Conclusion	Main Uncertainty	Evidence Maturity
Structure	covalent C_60_ cages form anisotropic 2D/few-layer lattices	phase labels not structurally universal	high (A–C)
Elasticity	qHP intrinsic stiffness ~10^2^ N m^−1^ and anisotropic	axis convention, thickness if GPa is used	high theoretical consistency after normalisation; no direct monolayer measurement
Strength/fracture	lower and more T-sensitive than ideal graphene; junctions govern failure	potential, T, strain rate, size, defects	medium–high computational support; no direct monolayer measurement
Electronic	experimentally anchored qHP is semiconducting; topology/stacking/field/impurity can tune the response	gap definition, topology and XC/many-body method	high qualitatively
Optical	strong excitons and polarisation anisotropy	screening/slab convention and layer count	medium–high theoretical consistency; limited direct validation
Thermal	strongly topology/direction/layer dependent	large unresolved model discrepancy in κ	medium; layered experimental baseline, unresolved monolayer conflict
Tribology	multicontact surfaces can support very low corrugation/superlubricity	finite-size, defects, real contact, experiment	medium–low; computational
Applications	catalysis, membranes and photodetection experimentally demonstrated	device stability, scale-up, phase reproducibility	rapidly increasing; result-level A/B distinctions required

## Data Availability

No new primary experimental or computational data were generated in this study. All source values were taken from the cited publications. The thickness-independent quantities used for cross-study benchmarking were calculated by the authors from those values; the method, inputs and inclusion status of each are given in Section 6.3 and Table 3.

## Abbreviations

The following abbreviations are used in this manuscript:

2D	two-dimensional
AIREBO	adaptive intermolecular reactive empirical bond order
AIREBO-M	AIREBO with a Morse-modified intermolecular term
AOR	ammonia oxidation reaction
BN	boron nitride
C60NET	covalent C60 network, as named by the cited authors
CBM	conduction band minimum
DFT	density functional theory
DFTB	density functional tight binding
EMD	equilibrium molecular dynamics
fcc	face-centred cubic
FE	Faradaic efficiency
GAP	Gaussian approximation potential
GenAI	generative artificial intelligence
GW-BSE	GW approximation with the Bethe–Salpeter equation
HER	hydrogen evolution reaction
HNEMD	homogeneous non-equilibrium molecular dynamics
HOMO	highest occupied molecular orbital
HSE	Heyd–Scuseria–Ernzerhof hybrid functional
LUMO	lowest unoccupied molecular orbital
MALDI-TOF	matrix-assisted laser desorption/ionisation time-of-flight
MD	molecular dynamics
MLIP	machine-learning interatomic potential
MTP	moment tensor potential
NEP	neuroevolution potential
NIR	near-infrared
NIR-II	second near-infrared window
NPs	nanoparticles
OER	oxygen evolution reaction
ORR	oxygen reduction reaction
PBE	Perdew–Burke–Ernzerhof exchange–correlation functional
qHP	quasi-hexagonal phase
qTP	quasi-tetragonal phase (qTP1, qTP2: specific variants)
ReaxFF	reactive force field
RP	a further network pattern label used by the cited authors
TM	transition metal
TP	tetragonal phase
TP2	a further tetragonal pattern label used by the cited authors
VBM	valence band maximum
VOC	volatile organic compound
XC	exchange–correlation

## Appendix A. Core C_60_-Network Literature Matrix (Verified Scope Through to 9 September 2026)

Table A1 is designed as a working corpus rather than a ranking. ‘Core’ means that the paper directly studies covalently connected C_60_ networks or a derivative whose behaviour depends on that network. Reviews are retained as mapping resources; isolated-C_60_ and fullerite papers are omitted from the matrix unless needed as a benchmark in the main text. The class refers to evidential proximity as defined in Section 2.2, not to a hierarchy of computational methods.

**Table A1:** Working corpus of core C_60_-network studies. The class refers to evidential proximity as defined in Section 2.2, not to a hierarchy of computational methods.

Year	Study	Method	Primary Contribution	Class
2022	Hou et al. [3]	Experiment	monolayer qHP synthesis; transport	A
2022	Tromer et al. [16]	DFT	electronic/optical/mechanical qHP	C
2022	Yu et al. [17]	DFT	electronic/optical/mechanical/thermoelectric	C/D
2022	Ribeiro Junior et al. [43]	ReaxFF MD	thermal stability; fracture	C
2022	Ying et al. [18]	DFT + NEP MD	anisotropy; strength; fracture	C
2022	Peng [61]	DFT/hybrid	photocatalytic water splitting	C/D
2022	Mortazavi & Zhuang [52]	MLIP-MD	strength; thermal conductivity	C
2023	Meirzadeh et al. [4]	Experiment	few-layer covalent network; thermal baseline	A
2023	Peng [13]	DFT	thermodynamic/dynamic/mechanical stability	C/D
2023	Shen et al. [19]	DFT	qHP elastic tensor	C
2023	Argaman & Makov [20]	DFT	structure, stability, electronic properties	C
2023	Zhao et al. [21]	Theory/atomistic	directional mechanical response	C
2023	Mortazavi [15]	DFT + MLIP	broad hypothetical C_60_ network design space	D
2023	Li et al. [81]	Theory	thermoelectric properties	C/D
2023	Dong et al. [53]	NEP-MD	high anisotropic thermal conductivity	C
2023	Miao et al. [69]	DFT	electric-field tuning	C/D
2023	Chang et al. [96]	DFT	VOC sensing and B-doping	C/D
2023	Wang et al. [84]	Experiment	few-layer pure-water photocatalysis	A
2023	Yu et al. [12]	MD	graphene support/encapsulation	C
2023	Li & Sun [60]	DFT	bulk/monolayer band structure and anisotropic optics	C/D
2023	Tong et al. [93]	CG-driven MD	monolayer fullerene membranes for H_2_ separation	D
2023	Ren et al. [94]	DFT + MD	hydrogen storage in monolayer C_60_ networks	D
2023	Jones & Peng [65]	hybrid DFT	2D-to-1D dimensionality benchmark for photocatalysis	D/E
2024	Ajori et al. [50]	MD	temperature/defect/size tensile response	C
2024	Giannopoulos [54]	ReaxFF MD	thermomechanical fracture	C
2024	Giannopoulos et al. [51]	ReaxFF/AIREBO-M MD	force-field benchmark	C
2024	Capobianco et al. [68]	electronic-structure + ET theory	polaron localisation and mobility	C
2024	Champagne et al. [75]	GW-BSE	excitons; anisotropic absorption	C
2024	Wei et al. [77]	DFT/optics	optical anisotropy	C/D
2024	Wang et al. [70]	DFT	stress-tunable anisotropic optoelectronics	C/D
2024	Yang et al. [79]	MD	monolayer/bilayer phonon thermal transport	C
2024	Liu et al. [57]	MD	interlayer friction	C
2024	Ying, Hod & Urbakh [55]	DFT/registry	structural superlubricity	C
2024	Chen et al. [91]	Experiment	organic-solvent membranes	B
2024	Zhang et al. [85]	Experiment	Bi/C_60_ electrocatalytic CO_2_ reduction	B
2024	Cassiano et al. [67]	Holstein–Peierls dynamics	large-polaron charge transport; strong anisotropy	C
2024	Yadav [24]	DFT	BN-doped qTP2 electronic/optical/transport tuning	D
2024	Yadav et al. [25]	DFT + classical	BN-doped qHP electronic/optical/mechanical response	D
2024	Chernozatonskii & Kochaev [64]	DFT	C_60_-polymer/h-BN electronic and optical heterostructure	C/D
2024	Suo et al. [71]	DFT	TM@qHP spin-sponge mechanism for O_2_ activation/CO oxidation	D
2024	Xu et al. [72]	real-time DFTB	photoinduced electron-nuclear dynamics in solvated qHP C_60_	C
2025	Zhang et al. [39]	Experiment	electrochemical exfoliation; photodetectors	A/B
2025	Shearsby et al. [62]	DFT/many-body	bilayer electronic/optical tuning	C
2025	Yan et al. [14]	DFT	five C_60_ network patterns	C/D
2025	Khan et al. [26]	spin-DFT	impurity-engineered spin functionality	D
2025	Hu et al. [82]	MTP-MD	topology-controlled thermal conductivity	C/D
2025	Shaikh et al. [23]	first-principles	anisotropic positive/negative thermal expansion	C/D
2025	Alekseev et al. [44]	GAP-MD	high-temperature stability benchmark	C
2025	Wang et al. [63]	DFT	trilayer sliding ferroelectricity	C/D
2025	Xu et al. [30]	DFT	Cu4 anchoring at intrinsic holes	C/D
2025	Huang et al. [29]	Experiment	Ir/monolayer HER catalyst	B
2025	Wang et al. [28]	Experiment	aryl functionalisation; N_2_ photoreduction	B
2025	Qiu et al. [58]	MD	mechanical + tribological response	C
2025	Peng & Pizzochero [7]	Review	first-principles perspective	E
2025	Wang et al. [8]	Review	broad 2D fullerene overview	E
2025	Kayley & Peng [32]	DFT/hybrid	pressure-derived layered C_60_ structural/electronic/optical benchmark	D/E
2025	Chen et al. [86]	Experiment	Pt/polymeric C_60_ network for ammonia oxidation	B
2025	Chen et al. [87]	Experiment	Ir/C_60_NET electron-buffering OER	B
2025	Wang et al. [88]	Experiment	Ru/few-layer 2D-C_60_ electron-buffering alkaline HER	B
2025	Wang et al. [89]	DFT + microkinetics	high-loading Cr@graphullerene ORR	D
2025	Peng & Pizzochero [66]	DFT	electronic structure of qHP/qTP-derived nanoribbons	D/E
2025	Wang et al. [95]	Experiment	Mg_4_C_60_ covalently bridged Li-ion anode framework	B/E
2026	Yuan et al. [40]	Experiment	alkali intercalation; rapid mono/bilayer exfoliation	A/B
2026	Alotaibi et al. [41]	Experiment	continuous-flow high-shear covalent sheets	A
2026	Yang et al. [56]	MD	graphullerene vdW heterostructure superlubricity	C
2026	Zhao et al. [45]	MD	intrinsic wrinkling and tensile morphology	C
2026	Yang et al. [80]	MD	overlapping-interface thermal transport; vacancies	C
2026	Lv et al. [31]	Experiment	phase-dependent NIR-II photothermal therapy	A/B
2026	Wang et al. [90]	Experiment	cobaloxime/2D C_60_ photocatalytic CO_2_ conversion	B
2026	Cai et al. [92]	Experiment	C_60_–graphene heterochannel membrane	B
2026	Lv et al. [9]	Review	covalent fullerene networks and catalysis	E
2026	Pereira Junior et al. [27]	DFT	endohedral N/Ce/Sr electronic and optical tuning	D
2026	Zhao, Guo & Qiu [59]	MD	1D Brownian motion of polymeric C_60_ nanoflakes on 2D crystals	C
2026	Paupitz et al. [10]	Review	2D carbon-allotrope review including fullerene networks	E
2026	Yadav [97]	DFT	BN-doped qTP H_2_O/CO_2_/N_2_ adsorption and HER screening	D
2026	Mei et al. [5]	Experiment	qHP-C_60_ sodium storage and Na–Cl_2_ conversion	B
2026	Jia et al. [6]	Experiment + DFT	coordination-triggered inter-cage σ-bond scission and disassembly	B/C
